# Polyhydroxy Hydrogel Electrolyte with In Situ Tuned Interface Chemistry for Ultra-Stable Biosensing-Compatible Zinc Batteries

**DOI:** 10.1007/s40820-025-02061-z

**Published:** 2026-01-26

**Authors:** Fengjiao Guo, Chunjiang Jin, Hongyu Mi, Ziqiang Liu, Bo Xu, Wenhan Jia, Guozhao Fang, Jieshan Qiu

**Affiliations:** 1 School of Chemical Engineering and Technology, Xinjiang University, Urumqi, 830017 People’s Republic of China; 2 School of Materials Science and Engineering, Key Laboratory of Electronic Packaging and Advanced Functional Materials of Hunan Province, Central South University, Changsha, 410083 People’s Republic of China; 3 State Key Laboratory of Chemical Resource Engineering, College of Chemical Engineering, Beijing, 100029 People’s Republic of China

**Keywords:** Hydrogel electrolyte, Polyhydroxy additive, Interface chemistry, Zinc batteries, Biosensing system

## Abstract

Aqueous zinc batteries (ZBs) represent a promising sustainable and safe energy storage technology, yet their widespread adoption is impeded by persistent interfacial instabilities at Zn anodes. This study reports a polyhydroxy hydrogel electrolyte (PASHE) with in situ regulated interface chemistry suitable for biosensing compatible ZBs. Benefiting from the well-integrated interface via in situ strategy, the hydroxyl-rich L-sorbose in PASHE establishes kinetically favorable Zn^2+^ transport pathways and regulates interfacial ion-adsorption hierarchies, synergistically homogenizing ion distribution and promoting preferential crystallographic orientation. Furthermore, PASHE constructs a low water-activity microenvironment via interfacial preferential adsorption, oxygen-rich solid electrolyte interphase evolution, and Zn^2+^ solvation sheath reconstruction. These effects enable Zn (002)-textured electrodeposition and inhibitory side reactions, achieving dendrite-free Zn plating/stripping with exceptional stability (3300 h in Zn//Zn cells) and near-perfect reversibility (average coulombic efficiency of 99.6% over 1200 cycles in Zn//Cu cells). This strategy delivers unprecedented cyclability in flexible Zn//I_2_ batteries (94.9% retention after 9000 cycles) and Zn-ion hybrid capacitors (98.0% after 43,000 cycles). Notably, we demonstrate an integrated biosensing platform that couples PASHE-based biosensor with cascaded Zn//I_2_ batteries, realizing real-time monitoring of physiological signals and biomechanical motions. This work proposes dual strategies of in situ approach and functional additive to design hydrogel electrolytes, bridging high-performance ZBs with next-generation biosensing technologies.

**Supplementary Information:**

The online version contains supplementary material available at 10.1007/s40820-025-02061-z.

## Highlights

- Polyhydroxy hydrogel electrolyte enables in situ dual regulations of Zn-electrolyte interfacial chemistry and bulk electrolyte properties.
- Reversible Zn anodes with exceptional cycling stability and perfect coulombic efficiency are achieved.
- A self-powered biosensing platform that integrates Zn//I_2_ batteries with hydrogel sensor achieves real-time physiological monitoring.

**Supplementary Information:**

The online version contains supplementary material available at 10.1007/s40820-025-02061-z.

## Graphical abstract

**Figure:**
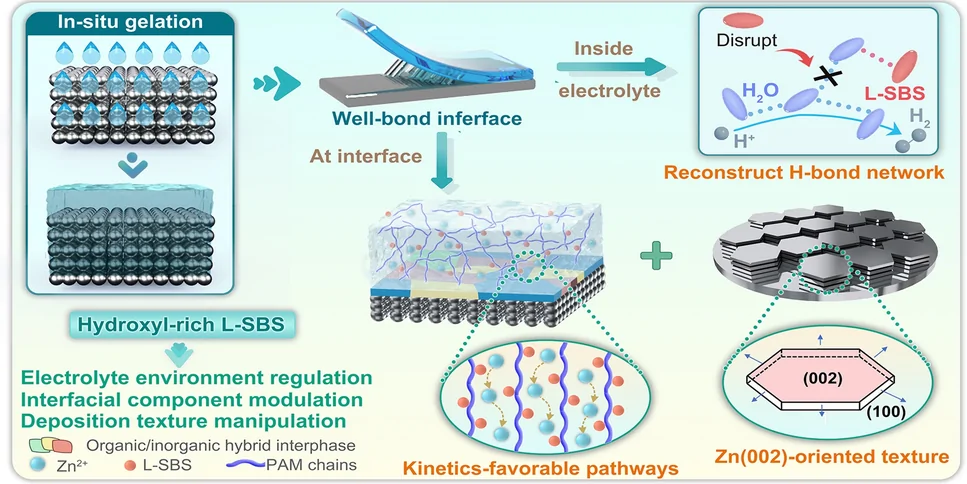

## Introduction

The global energy crisis and environmental degradation are urgently driving the development of sustainable energy storage technologies [1]. Among these, aqueous zinc batteries (ZBs) have emerged as a highly promising candidate, attracting considerable interest due to their inherent safety, cost-effectiveness, and straightforward manufacturing process [2, 3]. These advantages position ZBs as ideal power sources for next-generation wearable and implantable bioelectronics [4]. Nevertheless, the practical implementation of ZBs is still significantly impeded by critical challenges associated with the Zn anode, primarily uncontrollable dendrite growth and persistent parasitic side reactions [5]. These problems originate from the unstable the anode–electrolyte interface (AEI), which inevitably triggers rapid capacity decay, poor coulombic efficiency (CE), and even premature battery failure [6].

Substantial research efforts have been devoted to addressing the challenges of Zn anodes through various strategies, including artificial interfacial coatings, electrolyte engineering, and electrode structural design [7, 8]. Among these, electrolyte modulation has emerged as a pragmatic approach, owing to its cost-effectiveness and facile processing [9, 10]. For wearable devices that require direct human contact, the electrolytes must concurrently fulfill demands for flexibility and biocompatibility. In this regard, hydrogel electrolytes, featuring crosslinked polymer networks that minimize leakage risks, are inherently superior to other electrolyte systems [11, 12]. However, the immobile nature of conventional hydrogel electrolytes inevitably creates interfacial voids between the electrode and electrolyte, resulting in discontinuous ion transport pathways, elevated interfacial resistance, and localized Zn deposition at confined contact regions [11, 13]. Such drawbacks become especially critical under the repetitive mechanical deformation and electrochemical cycling inherent to flexible devices. Compounding these issues, the conventional ex situ fabrication method, which involves sandwiching preformed hydrogel electrolytes between electrodes, further exacerbates interfacial discontinuity and thereby accelerates anode degradation [14]. In contrast, in situ formation of hydrogel electrolytes directly on the electrode surface offers a compelling solution. This technique enables the creation of an adaptive and conformal electrode–electrolyte interface, effectively eliminating interfacial voids while promoting uniform ion transport and deposition. Importantly, this one-step integrated fabrication process bypasses the alignment and handling complexities associated with pre-formed components, significantly simplifying device assembly and enhancing structural integrity. Therefore, the in situ engineering approach is paramount for achieving practical and deformable energy storage devices. However, the functional interface chemistry required to empower such in situ hydrogel electrolytes remains extremely challenging [15]. Electrolyte additive engineering offers a good reference for this difficulty, as certain additives have been proven to reconstruct the double-layer structure (EDL) and form a robust solid electrolyte interphase (SEI) [16–18]. Despite these advances, the synergistical integration of in situ formed hydrogel electrolytes with functional biocompatible electrolyte additive, a critical step toward wearable applications [15, 19–21], has thus far remains largely unexplored.

Herein, we develop a polyhydroxy hydrogel electrolyte (PASHE) incorporating a biocompatible hydroxyl-rich L-sorbose (L-SBS) additive via in situ fabrication, aimed at effectively regulating interface chemistry of Zn anodes for wearable applications. Critically, the in situ gelation process, wherein liquid monomers polymerize while infiltrating the solid electrodes, enables the formation of a conformal and continuous electrolyte–electrode interface. This seamless integration maximizes the efficacy of L-SBS in modulating the AEI. Integrated theoretical computation and targeted experiments reveal that the L-SBS-modified hydrogel electrolyte establishes facile ion transport pathways, facilitating kinetically favorable and uniform Zn^2+^ migration, while regulating Zn^2+^ adsorption to induce oriented deposition with the exposed (002) plane. Furthermore, the hydroxyl-rich L-SBS, distributed both within the electrolyte and at the interface, reconstructs the inherent hydrogen-bond network, Zn^2+^ solvation sheath, and EDL, concurrently fostering the formation of an oxygen-rich hybrid SEI. These synergistic regulations establish a low-water-activity microenvironment, which enhances Zn corrosion resistance, suppresses HER, and homogenizes the interfacial ion/electrical field distributions. As a result, the Zn//Zn symmetric cell with PASHE achieves unprecedented cycling stability exceeding 3300 h at 0.5 mA cm^−2^/0.5 mAh cm^−2^, while the Zn//Cu asymmetric cell exhibits an ultrahigh average CE of 99.6% over 1200 cycles. When implemented in full cells, PASHE enables both Zn//I_2_ battery and zinc-ion hybrid capacitor (ZHC) to exhibit ultrastable performance, e.g., 94.9% retention after 9000 cycles for the Zn//I_2_ battery and 98.0% retention after 43,000 cycles for the ZHC. The self-powered sensing platform, which integrates three batteries and a PASHE-based strain sensor, enables real-time monitoring of physiological signals and human motions, thereby establishing a novel paradigm for next-generation biosensing healthcare systems.

## Experimental Section

### Preparation of Hydrogel Electrolytes

Firstly, 2 g acrylamide (AM) monomer was dissolved in 10 mL hot deionized water (60 °C), followed by dissolving 1.8 g L-SBS and 2.88 g ZnSO_4_·7H_2_O under stirring. After cooling for 1 h, 0.06 g ammonium persulfate (APS, as the initiator) and 0.006 g *N,N'*-methylenebisacrylamide (MBAA, as the cross-linker) were added to the above solution and stirred for 10 min. The resultant precursor solution underwent ultrasonic degassing (10 min) to eliminate bubbles before being precisely dispensed onto Zn foil substrates using a glass mold. Subsequent thermal polymerization at 60 °C for 90 min yielded in situ formation of ZnSO_4_-containing polyacrylamide/L-SBS hydrogel electrolyte (designated PASHE) conformally coated on the Zn surface. The concentration of L-SBS in PASHE was 1.0 M. For comparative analysis, a control electrolyte comprising ZnSO_4_-contained polyacrylamide hydrogel electrolyte (similarly denoted PAHE) was fabricated through identical processing parameters while omitting L-SBS incorporation. Additionally, hydrogel electrolytes with 0.5 and 1.5 M L-SBS were also prepared for comparison.

### Electrode Preparation and Device Assembly

#### Assembly of Zn//I_2_ Batteries

Firstly, an electrodeposition solution was prepared by mixing ZnI_2_ solution (10 mL, 0.5 M) and KI solution (10 mL, 0.5 M). Secondly, the electrodeposition was performed at 5 mV for 1200 s in a two-electrode system with Zn anode and AC cathode. Thirdly, the obtained I_2_ electrode was immersed in deionized water for 1200 s and dried at 40 °C for 12 h. Final device integration was achieved through in situ polymerization of hydrogel electrolytes at both electrode interfaces, establishing ion-conductive interphases to architecturally separate Zn anode and I_2_ cathode within hermetically sealed pouch cell configurations. The mass loading of active materials in the cathode was 2.6 mg.

#### Assembly of ZHCs

The activated carbon (AC) cathode was prepared by mixing AC, acetylene black, and polytetrafluoroethylene with a mass ratio of 8:1:1, dispersed in 5 mL of anhydrous ethanol by ultrasonication, dried at 70 °C, and cut into round sheets (a mass of 2 mg and an area of 1.1304 cm^2^). The hydrogel electrolytes were synthesized in situ between AC cathode and Zn anode (Zn foils with 100 μm thickness and 1.1304 cm^2^ area) to assemble quasi-solid-state ZHCs. The mass loading of active materials in the cathode was 1.6 mg.

## Results and Discussion

**Fig. 1:**
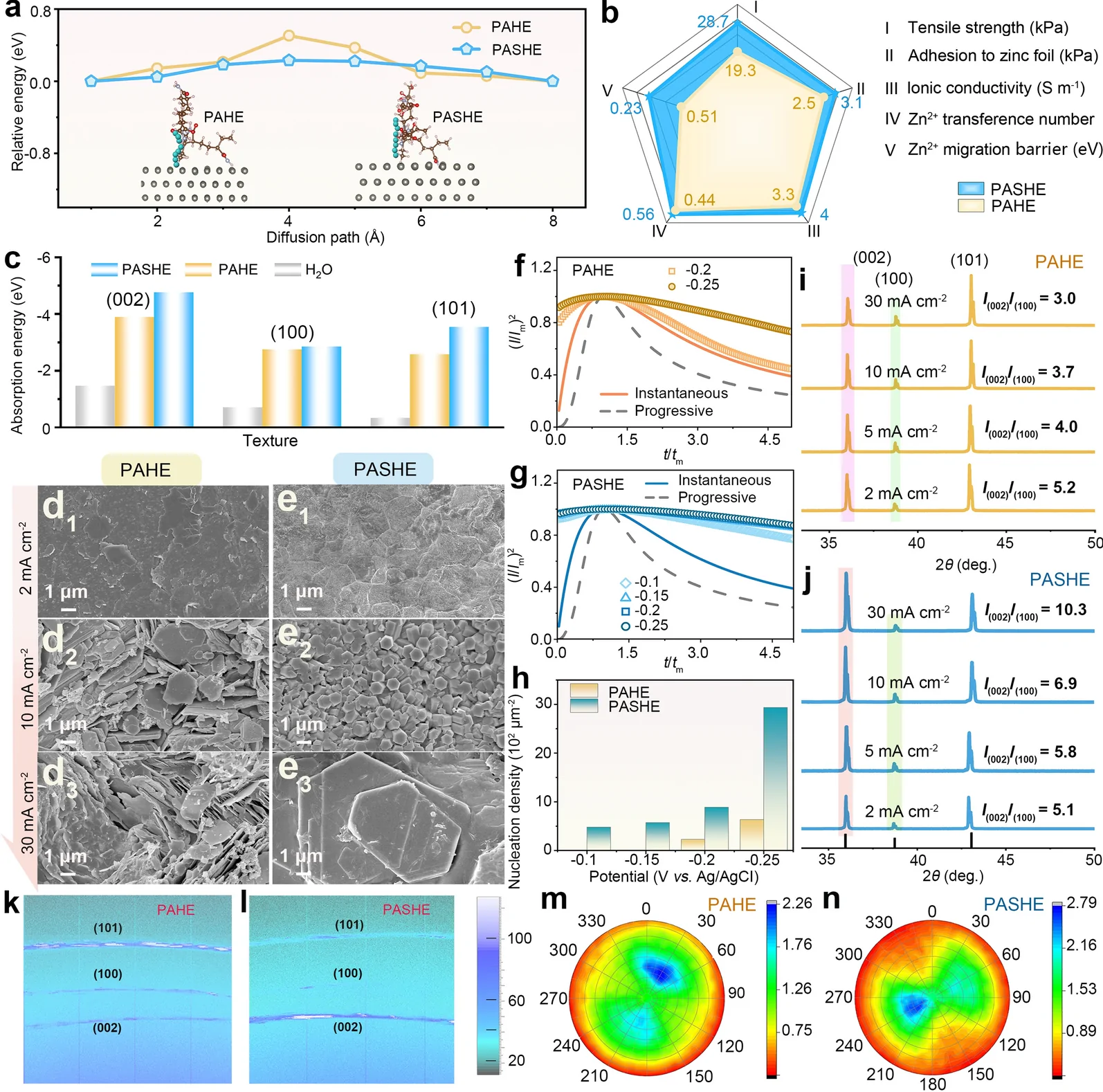
Mechanism investigation of L-SBS on regulating Zn^2+^ migration and deposition texture. **a** The diffusion paths and barriers of Zn^2+^ ions in PAHE and PASHE. **b** Comparisons of tensile strength, adhesive strength, ionic conductivity, Zn^2+^ transference number, and Zn^2+^ migration barrier between PAHE and PASHE. **c** Adsorption energies of H_2_O, PAHE, and PASHE on different planes of Zn metal. SEM images of Zn deposits for Zn//Zn cells with **d** PAHE and **e** PASHE after 100 cycles under different current densities from 2 to 30 mA cm^−2^. (*I*/*I*_m_)^2^–(*t*/*t*_m_) curves derived from the current response in CA plating using **f** PAHE and **g** PASHE. **h** Comparison of calculated nucleation densities. The corresponding XRD patterns of Zn deposits in **i** PAHE and **j** PASHE. 2D-GIXRD patterns of Zn deposits in **k** PAHE and **l** PASHE. XRD pole figures of Zn deposits in **m** PAHE and **n** PASHE after 100 cycles

The polyhydroxy-modified hydrogel electrolyte was fabricated directly on the zinc foil surface via an in situ polymerization strategy. This process entails free-radical polymerization of AM monomer in an aqueous electrolyte containing hydroxyl-rich L-SBS additive, initiated by APS and cross-linked by MBAA. An optimal L-SBS concentration of 1.0 M is identified, imparting the resultant PASHE with the highest ionic conductivity of 4.0 S m^−1^ and a maximum tensile strength of 28.7 kPa among the tested hydrogel electrolytes (Fig. S1). This combination of superior ionic conductivity and mechanical robustness makes PASHE particularly suitable for practical use in high-performance flexible devices, where efficient ion transport and durable deformation tolerance are essential. Notably, the in situ fabrication produces an integrated and seamless electrolyte–electrode interface without visible gaps, as evidenced by scanning electron microscopy (SEM) images (Fig. S2a). In contrast, the ex situ synthesized hydrogel electrolyte applied to the electrode exhibits a physically discontinuous interface with evident voids (Fig. S2b). Electrochemical impedance spectroscopy (EIS) quantitatively validates this difference in interfacial integrity. As depicted in Fig. S2c, the Zn/PASHE/Zn cell assembled via the in situ route exhibits substantially lower interfacial impedance than its ex situ counterpart, confirming its superior interfacial contact. This robust interface not only promotes efficient ion transport across the boundary but also fully harnesses the interface-regulating effect provided by the L-SBS additive. Furthermore, the incorporation of L-SBS markedly improves the interfacial adhesion of the hydrogel electrolyte. Adhesive strength tests reveal that PASHE adheres more strongly to zinc foil, copper foil, and graphite paper than the pristine polymer electrolyte PAHE, with measured strengths of 3.1 versus 2.5 kPa, 3.3 versus 2.3 kPa, and 4.8 versus 3.9 kPa, respectively (Fig. S3). The polymer networks functionalized with hydroxyl-rich L-SBS form a highly porous architecture (Fig. S4), creating favorable pathways for rapid Zn^2+^ migration. Moreover, PASHE exhibits improved Zn^2+^ transfer kinetics attributable to optimized solvation structures and migration pathways, as well as restricted SO_4_^2−^ mobility resulting from hydrogen-bond interactions between sulfate anions and hydroxyl groups of L-SBS [22, 23]. Consequently, the Zn^2+^ transference number in PASHE exceeds that in PAHE (0.56 vs. 0.44, Fig. S5). Density functional theory (DFT) simulations further support these findings, showing a significantly lower migration barrier of Zn^2+^ between PASHE and Zn anode (0.23 eV) compared to the PAHE system (0.51 eV) (Fig. 1a). Collectively, the L-SBS-modified hydrogel electrolyte demonstrates superior mechanical properties and enhanced Zn^2+^-transfer ability relative to the baseline system (Fig. 1b).

On the PASHE-anchored anode surface, Zn^2+^ exhibits stronger affinity to the Zn (101) and Zn (100) facets than to the Zn (002) plane (− 5.54 and − 4.67 vs. − 4.27 eV), as shown in Fig. S6. This trend suggests a relatively weak interaction between the Zn (002) facet with Zn^2+^ after PASHE modification. Theoretically, the crystal plane where Zn^2+^ ions are preferentially adsorbed, exhibits a faster growth rate, encouraging the exposure of other facets with low deposition rates [24]. The adsorption ability of Zn^2+^, H_2_O, and hydrogel electrolytes on the Zn surface was further investigated by DFT simulations. As displayed in Fig. 1c, the adsorption energies of PASHE on the (101), (100), and (002) facets of the Zn anode are − 3.54, − 2.85, and − 4.76 eV, respectively, which are significantly lower than those of PAHE (− 2.58, − 2.75, and − 3.89 eV) and H_2_O (− 0.34, − 0.71, and − 1.47 eV). These results indicate that the PASHE system leads to the lowest growth rate and the highest exposure of the Zn (002) facet, owing to the fact that the Zn^2+^ adsorption priority and the facet deposition rate jointly determine the crystallographic orientation of Zn deposition [25].

The crystallographic orientation/texture of cyclic products in different electrolytes were experimentally analyzed. After 100 cycles, the ex situ SEM images reveal that the PAHE-derived Zn surface displays flake-like deposits at 2 mA cm^−2^ (Fig. 1d_1_), and more flakes of various sizes scatter on the electrode surface at 5 mA cm^−2^ (Fig. S7a) and 10 mA cm^−2^ (Fig. 1d_2_). Upon increasing to 30 mA cm^−2^, the ridge-like flakes become the dominant morphology (Fig. 1d_3_). These dendritic deposits are bound to continued growth, ultimately leading to cell short-circuiting. Instead, the Zn anode cycled in PASHE presents more uniformly distributed Zn grains with more nucleation sites at 2 mA cm^−2^ (Fig. 1e_1_). As the current density increases, the (002) crystal plane expands, eventually producing a dense stack of horizontally aligned hexagonal platelets (Figs. 1e_2_, e_3_, and S7b). The nucleation behavior of Zn in pristine and L-SBS-modified hydrogel electrolytes were systematically investigated via transient analysis. The transient current profiles in PAHE and PASHE exhibit typical features of a diffusion-controlled Zn nucleation process (Figs. S8 and S9). In the chronoamperometry (CA) measurements, the current transient rise to a maximum current (*i*_max_), with the corresponding time (*t*_max_) reflecting the kinetics of Zn nucleation. By normalizing the current (*i*/*i*_max_) and time (*t*/*t*_max_) according to the Scharifker–Hills model, it is revealed that Zn nucleation in both electrolytes follows an instantaneous nucleation mode [26]. As compared in Fig. 1f, g, the CA curve in PASHE exhibits a plateau around *t*_max_ that remains stable over time, in contrast to the sharp peak observed in PAHE. This distinct profile suggests a more pronounced instantaneous nucleation behavior in PASHE [27]. Moreover, the nucleation density in PASHE is considerably higher than that in PAHE, indicating that the L-SBS additive effectively increases the active nucleation sites, thereby facilitating more uniform Zn deposition (Fig. 1h). X-ray diffraction (XRD) was employed to investigate the texture and crystalline structure of Zn deposits obtained after 100 cycles at different current densities. The XRD patterns show distinct peaks assigned to the Zn_4_(OH)_6_SO_4_·5H_2_O byproduct in the PAHE system, whereas no such peaks are observed in PASHE (Fig. S10) [28]. This result indicates that the side reactions are effectively suppressed in PASHE. Crystallographic texture quantification demonstrates a progressive Zn (002) plane dominance in PASHE, as evidenced by the orientation index (*I*_(002)_/*I*_(100)_) increasing from 5.1 to 10.3 (Fig. 1j). In contrast, the index of PAHE decreases from 5.2 to 3.0 over the current density range of 2−30 mA cm^−2^ (Fig. 1i).

Crystallographic interrogation via 2D grazing-incidence XRD (2D-GIXRD) and pole figure measurements reveals differences in Zn deposition textures between electrolyte systems. The 2D-GIXRD patterns exhibit a pronounced intensification of the Zn (002) Debye ring in PASHE (Fig. 1l), in contrast to PAHE (Fig. 1k), confirming that the L-SBS additive promotes preferential (002) plane orientation. From XRD pole figures, the distribution of the Zn (002) plane after cycling in PAHE is broad (Fig. 1m), whereas it is highly concentrated in PASHE (Fig. 1n). This discrepancy indicates the induction of a (002)-dominated crystallographic texture by the L-SBS-optimized electrolyte. This texture engineering directly governs the macroscopic uniformity of Zn deposition, as evidenced by in situ optical microscopy (Fig. S11). After 60 min of deposition in PAHE, uneven and loosely packed protrusions appear on the cross section of the Zn foil (top of Fig. S11). The continued electrodeposition exacerbates the morphological inhomogeneity, leading to conspicuous dendrite-like deposits after 120 min. Conversely, PASHE maintains planar Zn deposition throughout 120-min plating cycles, preserving electrode integrity via epitaxial propagation along the (002) basal plane (bottom of Fig. S11). This multiscale synergy between interfacial energetics and crystallographic preference in PASHE enables texture-controlled deposition, effectively suppressing dendrite formation and growth through fundamentally tailored electrodeposition kinetics.

**Fig. 2:**
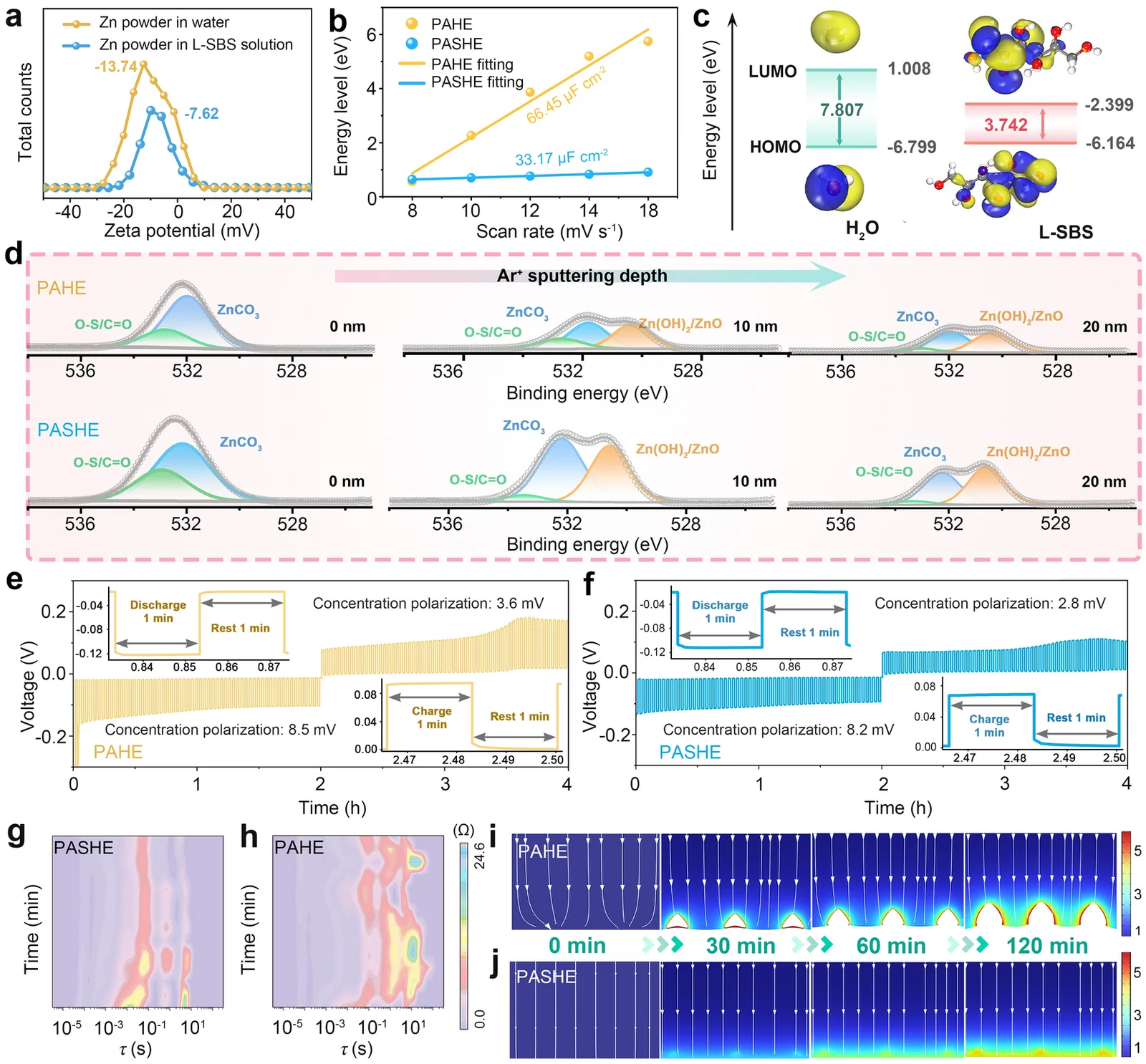
Investigation of interfacial components and behaviors. **a** Zeta potentials of zinc powder in water and L-SBS solution. **b** EDL measurements for Zn substrates in PAHE and PASHE. **c** HOMO and LUMO energy levels of H_2_O and L-SBS. **d** In-depth XPS analysis of O 1*s* on the Zn anode after 100 cycles in PAHE and PASHE. GITT curves of Zn//Zn symmetric cells in **e** PAHE and **f** PASHE. DRT results derived from EIS curves during Zn deposition in **g** PASHE and **h** PAHE. Finite element modeling of Zn^2+^ concentration field distributions and deposition morphology evolution in **i** PAHE and **j** PASHE during electrodeposition

The modulation of L-SBS for the interfacial component was further elucidated. Zeta potential measurements indicate a significant positive shift from −13.74 mV (bare Zn) to −7.62 mV upon L-SBS adsorption (Fig. 2a), demonstrating strong electrostatic interactions between the polyhydroxy additive and Zn surface [29]. The EDL results further confirm this judgment, where the EDL capacitance decreases dramatically from 66.45 mF cm^−2^ in PAHE to 33.17 mF cm^−2^ in PASHE (Figs. 2b and S12) [30]. These results suggest that L-SBS is favorable to excluding interfacial water molecules and reconstructing EDL, thereby inhibiting water-induced side reactions [31]. During the electrochemical process, the L-SBS molecules adsorbed on the Zn surface can decompose to participate in the construction of SEI, effectively regulating interfacial physicochemical behaviors. The propensity of L-SBS to decompose on the electrode surface was evaluated via molecular orbital level calculations. As summarized in Fig. 2c, the lowest unoccupied molecular orbital (LUMO) energy of L-SBS (−2.399 eV) is considerably lower than that of H_2_O (1.008 eV), demonstrating a higher tendency for L-SBS to accept electrons and participate in SEI formation [32, 33]. The structure and composition of the in situ formed SEI were investigated by X-ray photoelectron spectroscopy (XPS) with various sputtering times. The detailed XPS spectra of Zn electrodes cycled in PASHE and PAHE are depicted in Figs. 2d and S13. Prior to sputtering, the SEI surface in PASHE exhibits characteristic signals of organic and inorganic species in C 1*s*, O 1*s*, and S 2*p* spectra (Figs. 2d and S13a). Initial surface analysis (0 s sputtering) identifies existing organic components through C 1*s* (C=O: 288.6 eV, C−N/C−O: 286.0 eV, C−C/C−H: 284.8 eV) and O 1*s* (O–S/O–C: 532.8 eV) signatures, confirming the presence of L-SBS-derived organic constituents dominating the SEI periphery [15, 34]. Notably, the spontaneous sol–gel transition of the hydrogel electrolyte further enhances electrolyte-Zn interfacial adhesion, facilitating to enrich the organic-rich outer SEI with polymeric species [35]. Deconvolution of S 2*p* and O 1*s* spectra further confirms inorganic components such as ZnSO_4_, ZnSO_3_, and ZnCO_3_ [34, 36]. Thereinto, ZnCO_3_ acts as a Zn^2+^-conducting phase, elevating the interfacial energy, thus promoting Zn^2+^ transport and inhibiting dendrite formation [37, 38]. After sputtering for 35 s, the emergence of ZnS and Zn(OH)_2_/ZnO signals in S 1*s* and O 1*s* spectra indicates the co-precipitation of Zn^2+^ with sulfur-containing species and OH^−^. Such a SEI layer with sulfidic composites enhances interfacial stability and suppresses side reactions [39]. Notably, depth profiles reveal a gradient SEI architecture: organic components diminish as Ar^+^ sputtering proceeds, whereas inorganic species persist into deeper regions, forming an inorganic-dense subsurface. Comparative quantitative XPS analysis further reveals a higher content of oxygen-containing organics in PASHE-derived SEI relative to PAHE (Figs. 2d and S13b), confirming L-SBS-mediated oxygen enrichment. These results are consistent with time-of-flight secondary ion mass spectrometry (TOF–SIMS) results. 3D rendering images (Figs. S14 and S15) highlight a more uniform and abundant distribution of O-containing species throughout the SEI formed in PASHE, supporting the formation of an O-enriched hybrid interphase. This tailored SEI structure promotes uniform Zn deposition and effectively shields the electrode from corrosion [40].

The oxygen-rich SEI with zincophilic components, synergistically coupled with the L-SBS-optimized Zn^2+^ transport network, collectively regulates the interfacial distribution of Zn^2+^ ions, thereby effectively mitigating concentration polarization. Quantitative evaluation of concentration polarization in PASHE and PAHE was performed using galvanostatic intermittent titration technique (GITT). The concentration polarizations during both electroplating (2.8 vs. 3.6 mV) and stripping (8.2 vs. 8.5 mV) processes are reduced in PASHE (Fig. 2f) relative to PAHE (Fig. 2e). Further elucidation of Zn^2+^ interfacial kinetics was achieved through the distribution of relaxation times (DRT) analysis. As illustrated in Fig. 2g, h, the characteristic peaks located at relaxation times (*τ*) of approximately 10^−3^−10^−2^, 10^−2^−10^−1^, 10^−1^−10^0^, and 10^0^−10^1^ s are assigned to the adsorption (*R*_ads_), migration (*R*_mig_), charge transfer (*R*_ct_), and diffusion (*R*_diff_) processes of Zn^2+^ ions, respectively [41]. Notably, all characteristic peaks for the L-SBS-modified PASHE (Figs. 2g and S16) exhibit reduced values and intensities in *τ* compared with PAHE (Figs. 2h and S16), reflecting more facile adsorption, migration, charge transfer, and diffusion of Zn^2+^ within the L-SBS-modified hydrogel electrolyte. These observations demonstrate the accelerated interfacial kinetics enabled by the L-SBS-tailored EDL and SEI, which promotes more uniform Zn plating/stripping behavior and consequently contributes to the suppressed dendrite growth [42]. The modulation mechanism of L-SBS on Zn deposition was investigated by finite element simulations. Initially, Zn^2+^ ions selectively adsorb on certain sites of the electrode surface due to the absence of L-SBS, as reflected by the uneven Zn^2+^ concentration field and electric field distributions at 0 min in Figs. 2i and S17a. As electrodeposition proceeds, the Zn^2+^ ions become enriched around protrusions, exacerbating the inhomogeneity of the electric field and Zn^2+^ concentration field distributions, which results in the formation and growth of dendrites. In stark contrast, L-SBS-functionalized interfaces enable homogenizations of interfacial electric field and ion flux during the deposition process (Figs. 2j and S17b). Collectively, these results demonstrate that L-SBS facilitates the formation of a gradient organic–inorganic hybrid SEI with oxygen-rich characteristics, which effectively mitigates concentration polarization, accelerates Zn^2+^ interfacial kinetics, and homogenizes ion deposition, thereby significantly suppressing Zn dendrite formation.

**Fig. 3:**
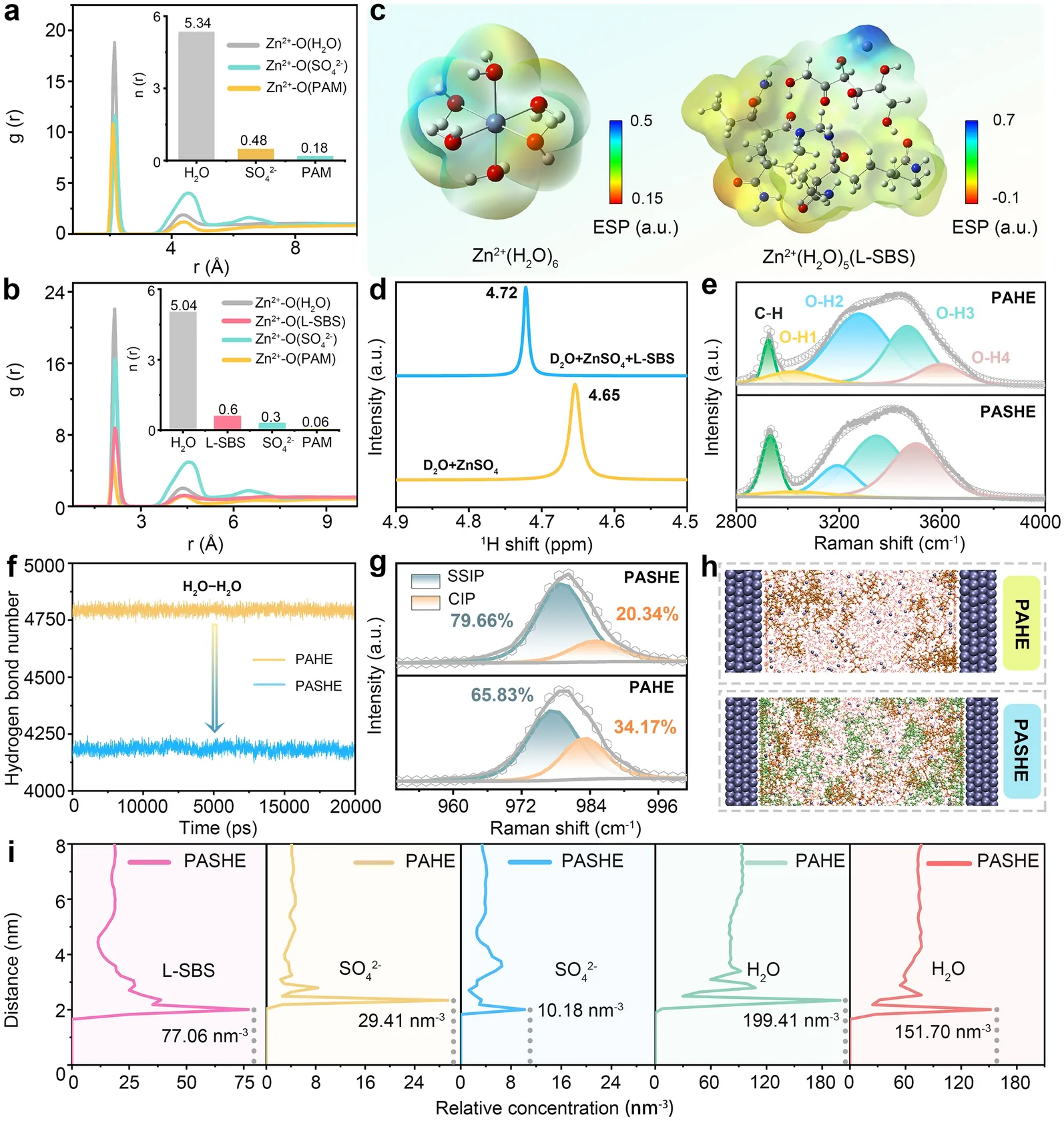
Electrolyte and interfacial microenvironment modulation. RDFs and coordination numbers of Zn^2+^−O in **a** PAHE and **b** PASHE calculated from MD simulations. **c** ESP maps of Zn^2+^(H_2_O)_6_ and Zn^2+^(H_2_O)_5_(L-SBS). **d** ^1^H NMR spectra of ZnSO_4_ solutions with and without L-SBS. **e** Raman spectra of PAHE and PASHE for *v*(–OH). **f** Calculated H-bond numbers between water molecules in PAHE and PASHE. **g** Raman spectra of PAHE and PASHE for *v*(SO_4_^2−^). **h** Snapshots of Zn metal interface in PAHE and PASHE from MD simulations. **i** Corresponding interfacial distributions of L-SBS, H_2_O, and SO_4_^2−^

The evolution of Zn^2+^ solvation upon introducing L-SBS were probed using molecular dynamics (MD) simulations in conjunction with radial distribution functions (RDFs) and average coordination number. In the control system (PAHE), Zn^2+^ exists in a conventional solvation structure coordinated primarily by H_2_O (primary peak at 2.15 Å, coordination number = 5.34), SO_4_^2−^ (2.10 Å, 0.48), and polyacrylamide (2.15 Å, 0.18) (Figs. 3a and S18a). Remarkably, the introduction of L-SBS induces a marked reorganization of the Zn^2+^ solvation shell, as evidenced by the emergence of a distinct RDF peak at 2.14 Å corresponding to L-SBS coordination (Figs. 3b and S18b). Quantitative analysis demonstrates that L-SBS (coordination number = 0.6) partially displaces solvent molecules, reducing coordination numbers of H_2_O, SO_4_^2−^, and PAM to 5.04, 0.30, and 0.06, respectively. This structural rearrangement stems from the substitution of solvating H_2_O molecules with polyhydroxy L-SBS, effectively reconstructing the primary solvation environment of Zn^2+^. To further clarify the solvation effect of L-SBS, the electrostatic potential (ESP) maps were constructed to visualize the charge distribution in Zn^2+^ solvation models (Fig. 3c). Compared to the conventional Zn^2+^(H_2_O)_6_ configuration, the L-SBS-modified solvated structure shows an expanded region of negative potential, confirming that L-SBS can alleviate the surrounding repulsion of Zn^2+^ to reorganize a kinetically favorable solvation structure [43]. The decreased hydration number within the solvation sheath is advantageous for suppressing HER and mitigating Zn corrosion, thereby prolonging the cycle life of Zn anodes [44].

Conventional aqueous electrolytes inherently suffer from high water activity that promotes parasitic reactions. Polyhydroxy compounds can effectively mitigate this issue through hydrogen-bond-mediated water molecule confinement, as demonstrated by the comprehensive spectroscopic analyses and MD simulations. Fourier transform infrared (FTIR) spectroscopy reveals significant modifications in hydrogen-bonding networks (Fig. S19). The O–H bending (1600–1800 cm^−1^) and stretching (3000–3600 cm^−1^) vibrations exhibit distinct blue- and red-shifts, respectively, in PASHE compared to PAHE, indicating substantial weakening of inter-water hydrogen bonds [45]. ^1^H nuclear magnetic resonance (^1^H NMR) analysis (Fig. 3d) provides complementary evidence, with the proton resonance shifting downfield upon L-SBS incorporation, confirming the formation of stronger hydrogen bonds between water molecules and polyhydroxy additive [46]. Raman spectral deconvolution (Fig. 3e) quantifies these changes through four resolved components in the O–H stretching region (3000–3700 cm^−1^). The O–H1 and O–H2 modes correspond to the coupled vibrations between water molecules and the stretching vibrations involving hydroxyl groups and their nearest neighbors, while the O–H3 and O–H4 modes represent the O–H stretching vibrations of water molecules with broken hydrogen bonds [47]. After introducing L-SBS, the O–H1 and O–H2 modules exhibit obvious intensity decay, whereas the intensities of O–H3 and O–H4 increases significantly. This indicates that the H-bond interaction between water and L-SBS attenuates the intrinsic H-bond network of the aqueous solvent. The MD simulation results in Fig. 3f support this conclusion, showing fewer H-bond numbers in PASHE than PAHE. Furthermore, the *v*(SO_4_^2−^) Raman band at ~ 980 cm^−1^ undergoes a 4.2 cm^−1^ blue-shift in PASHE, accompanied by a significant redistribution of ion pair species (Fig. 3g). Quantitative analysis shows the solvent-separated ion pair (SSIP) population increases from 65.83 to 79.66%, while contact ion pairs (CIP) decrease from 34.17 to 20.34% [48], indicating reduced SO_4_^2−^ participation in Zn^2+^ inner solvation shells.

Beyond the inherent properties of the electrolyte, the interfacial microenvironment is also effectively modulated by L-SBS, as evidenced by MD simulations. Representative MD snapshots are provided in Fig. 3h. As depicted in Fig. 3i, L-SBS molecules accumulate densely within an interfacial layer of approximately 1−2 nm, displacing interfacial H_2_O and SO_4_^2−^ species, which in turn suppresses HER and mitigates by-product accumulation. Together, these findings demonstrate that the L-SBS-modified electrolyte significantly reduces the populations of coordinated, free, and interfacial water molecules, thus effectively inhibiting water-induced side reactions.

**Fig. 4:**
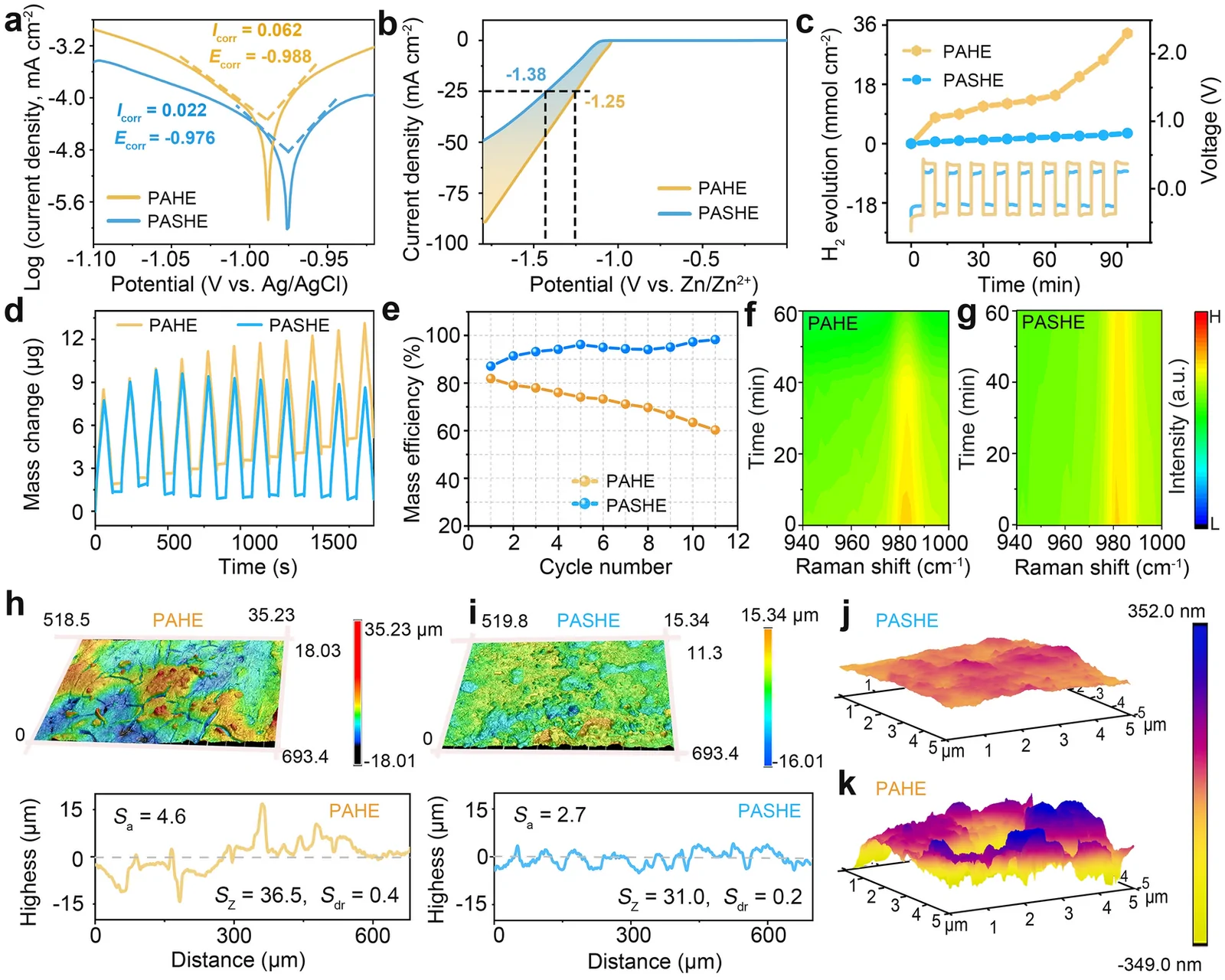
Inhibitory effect on interfacial side reactions. **a** Tafel curves of PAHE and PASHE. **b** LSV curves of Zn electrodes in PAHE and PASHE. **c** in situ GC–MS analysis of hydrogen evolution in PAHE and PASHE. **d** Mass changes and **e** mass efficiencies of Zn electrodes during plating/stripping in PAHE and PASHE. Change of Raman intensity during Zn deposition in **f** PAHE and **g** PASHE. The CLMS images of Zn electrodes cycling for 100 h in **h** PAHE and **i** PASHE. AFM images of Zn electrodes after cycling for 100 h in **j** PASHE and **k** PAHE

The inhibitory effect of PASHE on side reactions was systematically investigated. As shown in Tafel curves (Fig. 4a), the corrosion potential of the Zn electrode in PASHE increases from −0.988 to −0.976 V compared to that in PAHE, suggesting the restrained corrosion tendency in PASHE. Moreover, a lower corrosion current density of 0.022 mA cm^−2^ is determined for the Zn electrode in PASHE than the one in PAHE (0.062 mA cm^−2^), signifying the reduced Zn corrosion rate in the L-SBS-added hydrogel electrolyte [49]. Linear sweep voltammetry confirms enhanced HER suppression, with the overpotential at −25 mA cm^−2^ increasing by 130 mV in PASHE (−1.38 V) relative to PAHE (−1.25 V) (Fig. 4b). Quantitative in situ gas chromatography–mass spectrometry (GC–MS) analysis provides direct evidence of hydrogen evolution suppression, revealing that PASHE limits H_2_ generation to merely 9.6% of that observed in PAHE 3.2 vs. 33.5 mmol cm-^2^ after 100 h of operation (Fig. 4c). This result directly and strongly confirms the inhibitory ability of L-SBS additive on HER. The inhibitory effect of PASHE on side reactions was further supported by in situ electrochemical quartz crystal microbalance (EQCM), which can sensitively and accurately detect the mass change of the working electrode during Zn plating/stripping [50]. The control system (PAHE) exhibits progressive mass accumulation due to irreversible byproduct deposition [51], while PASHE maintains near-ideal mass balance throughout cycling (Fig. 4d). Quantitative analysis of mass efficiency also confirms these observations (Fig. 4e). PASHE sustains exceptional mass efficiency (> 90%) over extended cycling, whereas PAHE shows continuous deterioration, revealing fundamental differences in reaction reversibility [52].

The migration trajectories of ions at the electrode/electrolyte interface in Zn//Cu cells across different electrolytes were tracked by in situ Raman spectroscopy. During electroplating, Zn^2+^ and SO_4_^2−^ migrate toward Cu and Zn electrodes, respectively, driven by the electric field [53]. Raman spectra visualize the concentration change of SO_4_^2−^ anions at the Zn electrode, which directly reflects the concentration change of Zn^2+^ ions near the Cu electrode, as illustrated in Fig. S20. In PAHE (Fig. 4f), the SO_4_^2−^ signal intensity decays rapidly after 40 min plating, declaring the inhomogeneous Zn^2+^ flux that likely induces pronounced concentration polarization near the electrode–electrolyte interface [30]. Remarkably, PASHE maintains a nearly constant SO_4_^2−^ spectral intensity throughout plating, signifying a stable and uniform Zn^2+^ flux (Fig. 4g). This observation discloses that L-SBS adsorbs on the electrode surface to effectively regulate the interfacial ion distribution, thereby facilitating uniform Zn deposition, which agrees with the GITT analysis. The morphological differences in Zn deposits between PAHE and PASHE were examined by confocal laser scanning microscopy (CLSM) and atomic force microscopy (AFM) detections. In terms of the CLSM images in Fig. 4h, more rough surface appearance with high roughnesses was observed for the Zn electrode cycled in the baseline electrolyte, including arithmetic mean height (*S*_a_) of 4.6 μm, maximum height (*S*_z_) of 36.5 μm, and developed interfacial area ratio (*S*_dr_) of 0.4. In contrast, the Zn electrode cycled in the L-SBS-containing hydrogel electrolyte exhibits smoother surface texture with lower roughnesses (*S*_a_ = 2.7 μm,* S*_z_ = 31.0 μm, and *S*_dr_ = 0.2; Fig. 4i). Correspondingly, the AFM image reveals a relatively flat and compact Zn surface with low roughness after 100 h of cycling in PASHE (Fig. 4j), in stark contrast to the bumpy surface topography observed in PAHE (Fig. 4k). This distinct morphological evolution directly corroborates the role of L-SBS in guiding uniform Zn deposition and mitigating parasitic reactions.

**Fig. 5:**
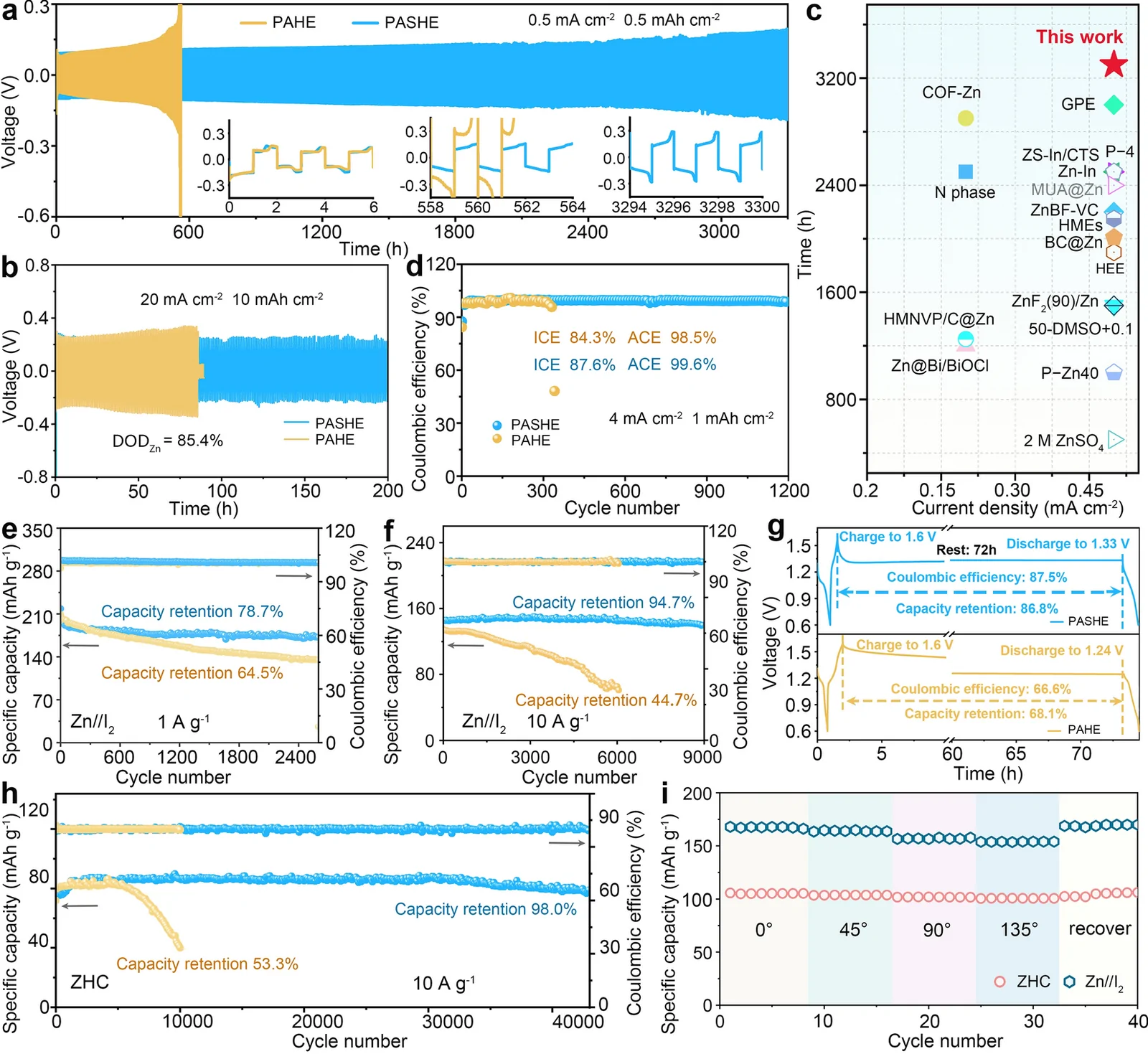
Performances of Zn plating/stripping and full cells. Cycling performance of Zn//Zn cells using PAHE and PASHE at **a** 0.5 mA cm^−2^/0.5 mAh cm^−2^ and **b** 20 mA cm^−2^/10 mAh cm^−2^. **c** Comparison of cycling performance between PASHE-based Zn//Zn cell with recently reported results. **d** CE values of Zn//Cu cells using PAHE and PASHE. Cycling stability of Zn//I_2_ batteries using PASHE and PAHE at **e** mA g^−1^ and **f** 10 mA g^−1^. **g** Voltage–time profiles of Zn//I_2_ batteries using PAHE and PASHE. **h** Cycling stability of ZHCs using PASHE and PAHE. **i** Specific capacities of the PASHE-based Zn//I_2_ and ZHC devices under bending deformations

The reliability of L-SBS in enhancing the electrochemical stability and reversibility of Zn anodes was assessed by galvanostatic cycling measurements of Zn//Zn and Zn//Cu cells. Our observation reveals that, under 0.5 mA cm^−2^/0.5 mAh cm^−2^, the Zn//Zn cell utilizing L-SBS-free PAHE exhibits progressively increasing voltage polarization with notable fluctuations after approximately 300 h of cycling. Subsequently, a sudden failure occurs and a catastrophic surge in polarization after 561 h (Fig. 5a). This failure stems from serve internal electrode passivation caused by the byproduct accumulation and dendrite proliferation [54]. Unlike that, the cell equipped with PASHE achieves exceptional operation stability with an ultra-long service lifetime of 3300 h. Even at a higher current density of 2 mA cm^−2^ and areal capacity of 2 mAh cm^−2^, the symmetric cell with the L-SBS-optimized hydrogel electrolyte maintains stable for 1700 h, representing a threefold elongation in the lifespan compared to the cell using the blank hydrogel electrolyte (Fig. S21). Furthermore, the cell’s performance was examined under a high depth of discharge (DOD_Zn_), as shown in Fig. 5b. At a high DOD_Zn_ of 85.4% (20 mA cm^−2^ and 10 mAh cm^−2^), the PASHE-based Zn//Zn cell achieves a cycling life of 200 h with a stable voltage distribution, surpassing the short-lived cell with PAHE (85 h). This life extension indicates effective suppression of dead zinc accumulation in PASHE at high zinc utilization. The beneficial influence of L-SBS further extends to the rate capability under elevated current densities. As presented in Fig. S22, the Zn//Zn cell with the L-SBS-modified PASHE displays a steady and regular variation in performance as the current density increases from 1 to 50 mA cm^−2^. However, the symmetric cell employing PAHE shows a sharp increase in voltage polarization when the current density reaches 30 mA cm^−2^, followed by complete failure at 50 mA cm^−2^. These results validate that the L-SBS in PASHE can greatly improve the high-rate durability of the cell. In addition, the cycling ability of the Zn electrode in PASHE is benchmarked against many state-of-the-art systems based on electrolyte, electrode, and separator modification strategies, highlighting the superior effectiveness of L-SBS in stabilizing Zn electrochemistry (Fig. 5c, Table S1).

Quantitative assessment of electrochemical reversibility via Zn//Cu cell cycling reveals the fundamental improvements achieved through L-SBS additive engineering. The Zn//Cu cell using PAHE shows compromised cycling reversibility, as reflected by its low initial CE (ICE, 84.3%), a moderate average CE (ACE, 98.5%), and a limited cycle life of only 341 cycles (Fig. 5d). These performance constraints are attributed to pronounced side reactions occurring at the AEI without L-SBS protection. Comparatively, the asymmetric cell incorporating PASHE delivers a high ICE of 87.6%, which rapidly increases and stabilizes at high values, sustaining stable operation for over 1200 cycles and harvesting an ultra-high ACE of 99.6%. Capacity–voltage profiles in Fig. S23 show that the PASHE-based cell displays highly overlapping voltage curves at selected cycles, contrasting sharply with the disordered voltage evolution observed in the PAHE-based system. The significantly improved ACE, together with highly overlapping voltage profiles, demonstrates enhanced reversibility of Zn plating/stripping. This improvement originates from homogeneous Zn deposition and suppressed side reactions, both facilitated by the regulatory function of L-SBS. Such an inference is further supported by distinct morphological variations observed across different hydrogel electrolytes. As revealed by the SEM images (Fig. S24), the post-cycled Zn and Cu electrodes in PAHE display scaly dendritic and moss-like deposition morphologies, respectively. In contrast, PASHE enables the relatively uniform and flat surfaces, highlighting the beneficial role of L-SBS in promoting homogeneous electrodeposition. The enhanced plating/stripping stability was further quantified via a reservoir half-cell methodology [55, 56]. According to the equation for ACE illustrated in Fig. S25, the cell with PASHE achieves a high value of 90.6%, compared to 81.3% for the contrast cell with PAHE. This multiscale validation, encompassing both symmetric cell longevity and asymmetric cell reversibility, establishes L-SBS as an effectual additive for achieving high-efficiency zinc electrochemistry through electrolyte and interfacial regulations.

Systematic evaluation of polyol-mediated regulation strategy was conducted through quasi-solid-state Zn//I_2_ pouch batteries. The successful fabrication of I_2_ cathode was analyzed by SEM and XRD (Fig. S26). The PASHE-based battery demonstrates excellent cycling stability, with capacity retentions of 88.6% (570 cycles) and 78.7% (2600 cycles) at 0.5 and 1 A g^−1^, respectively (Figs. S27 and 5e). These values are superior to the 57.5% (309 cycles) and 64.5% (2600 cycles) retentions achieved by the contrast cell using PAHE. At a high current density of 10 A g^−1^, the L-SBS-containing PASHE boosts the capacity retention to 94.7% over 9000 cycles (Fig. 5f). In contrast, the cell without L-SBS additive exhibits a significantly shorter lifespan of only 6100 cycles, accompanied by rapid capacity attenuation (44.7% retention) and fluctuating CE. The results underscore the enhanced cycling stability of full cells when using the L-SBS-containing hydrogel electrolyte. Notably, the cyclability of PASHE-enabled Zn//I_2_ battery surpasses those of many recently reports (Fig. S28 and Table S2). As a proof of concept, the practical viability of the PASHE-based Zn//I_2_ battery was evaluated by powering small appliances. The inset of Fig. S28 exemplifies the successful operation of an electronic timer and a mobile phone driven by single and three-in-series batteries, respectively. Moreover, three batteries in series are able to light optical-fiber model lamps. In terms of rate performance shown in Fig. S29, the battery utilizing PASHE shows high specific capacities of 255.1 mAh g^−1^ at 0.2 A g^−1^ and 189.1 mAh g^−1^ at 5 A g^−1^. This fact reflects a superior rate capability in comparison with the PAHE-based battery (74.1% vs. 68.4%), which provides 243.4 mAh g^−1^ at 0.2 A g^−1^ and 166.4 mAh g^−1^ at 5 A g^−1^. Moreover, the specific capacity in PASHE is recoverable upon returning to the initial current density, demonstrating the favorable electrochemical reversibility in this hydrogel electrolyte. Additionally, the self-discharge property of quasi-solid-state Zn//I_2_ batteries with different electrolytes was examined by monitoring the voltage decay of fully charged batteries during rest for 72 h to the complete discharge state. As demonstrated in Fig. 5g, the Zn//I_2_ battery using PAHE holds low CE (66.6%) and capacity retention (68.1%). Satisfyingly, the battery incorporating PASHE achieves significantly higher performance (87.5% and 86.8%). These results corroborate the effectiveness of the L-SBS additive in improving the availability of Zn-based batteries by restraining side reactions and byproduct generation [34]. In addition, the devised PASHE also performs well in a typically high-power ZHC system. The coal pitch-based carbon with abundant pores and high surface area is demonstrated (Fig. S30), which is suitable as the cathode of the ZHC device. As depicted in Fig. 5h, the quasi-solid-state pouch ZHC with PASHE displays an exceptional cycling lifespan of 43,000 cycles, accompanied by an ultra-high capacity retention of 98.0% at 10 A g^−1^. However, its counterpart suffers a rapid capacity fade only after 5300 cycles, with a low retention of 53.3% after 10,100 cycles. The differing cycle lives of these devices reflect the effect of L-SBS on promoting the device stability, as visually corroborated by the SEM observation. As illustrated in Fig. S31, the Zn anode remains dendrite-free surface after cycling in PASHE, substantiating the validity of the L-SBS in stabilizing Zn anodes. In addition, the PASHE-based ZHC delivers a specific capacity of 161.6 mAh g^−1^ at 0.2 A g^−1^ with 44.4% retention of initial capacity (71.8 mAh g^−1^ at 30 A g^−1^) (Fig. S32). More notably, both the quasi-solid-state Zn//I_2_ and ZHC pouch cells exhibit remarkable mechanical flexibility, showing negligible capacity variations under bending angles of 0°, 45°, 90°, and 135° (Figs. 5i, S33, and S34). This excellent bendability stems from the superior mechanical toughness of the L-SBS-optimized hydrogel electrolyte and the stable electrode–electrolyte interface, which collectively allow the flexible devices to endure repeated mechanical deformations typical of daily wearable use. Overall, this validation across multiple device configurations confirms PASHE as a promising hydrogel electrolyte for advanced Zn-based energy storage systems, particularly in flexible and wearable applications.

**Fig. 6:**
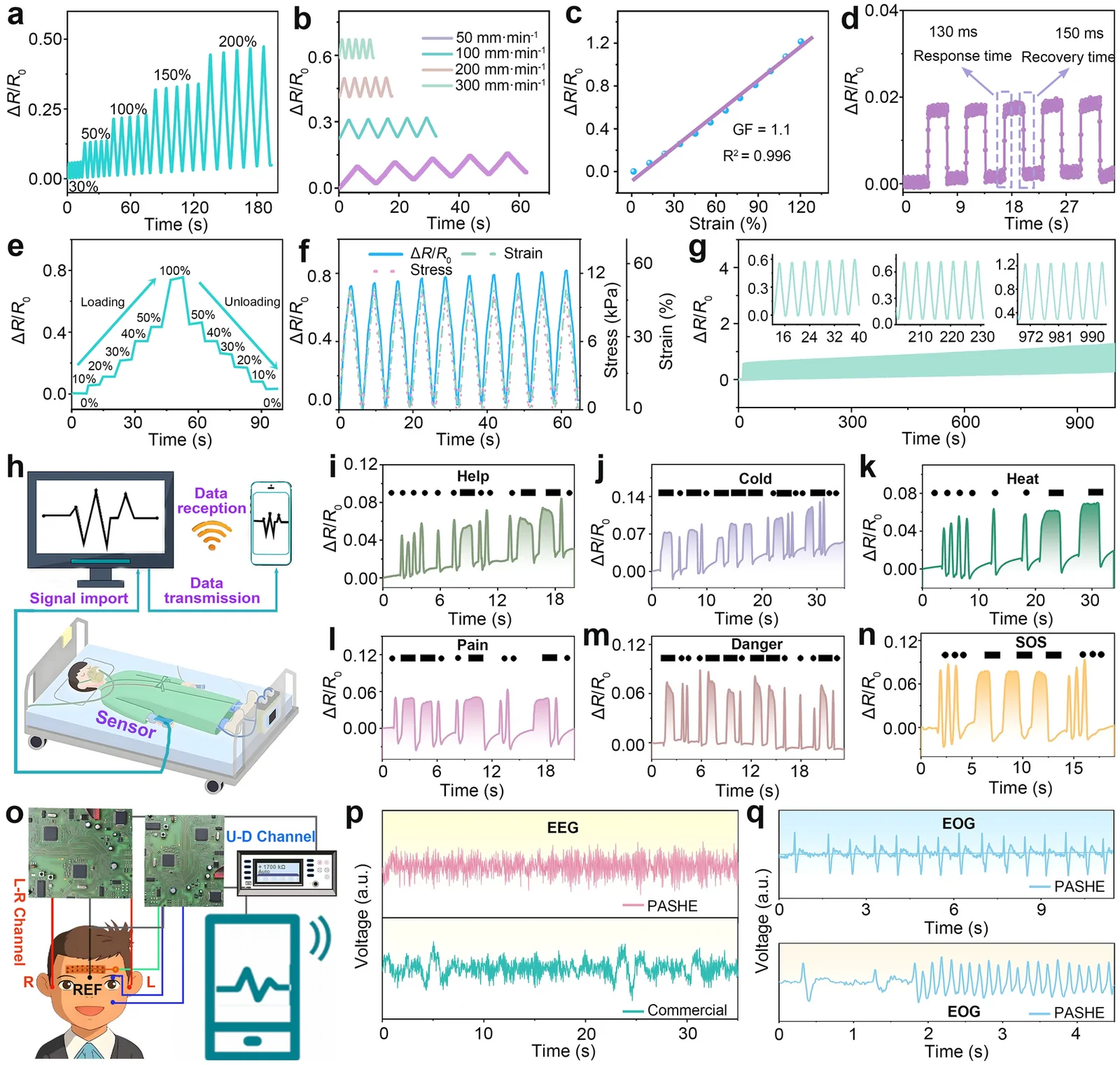
Sensing performance of the PASHE-based sensor. **a** Dynamic electrical responses of the strain sensors under the strain of 30%, 50%, 100%, 150%, and 200%. **b** Dynamic electrical responses under different testing speed from 50−300 mm min^−1^ at the strain of 100%. **c** Relative resistance change over a strain of 0−130% and the corresponding GF of the strain sensor. **d** Response time of the strain sensor upon the quickly bending of wrist. **e** Δ*R*/*R*_0_ of the PASHE-based sensor upon responding the stepwise strain (0–100%). **f** Consistency of Δ*R*/*R*_0_ (blue solid line), corresponding stress (pink dash line), and strain (green dotted line) for the PASHE-based sensor. **g** Δ*R*/*R*_0_ of the strain sensor under 60% for 500 cycles without any resting time at the speed of 50 mm min^−1^. **h** Schematic diagram of sign language recognition system with the PASHE-based sensor. Language identification of the PASHE-based sensor in response to finger motions: **i** "Help", **j** "Cold", **k** "Heat", **l** "Pain", **m** "Danger", and **n** "SOS" in Morse Codes. **o** Schematic diagram of neurophysiological monitoring using PASHE-based sensor. **p** EEG signal recorded by PASHE-based and commercial sensors. **q** EOG signals recorded by the PASHE-based sensor

The skin compatibility of PASHE was first assessed via wearing tests conducted over 0.5, 1, and 3 h (Fig. S35a). Notably, the skin region in contact with PASHE shows no evidence of irritation or itching, indicating its good biocompatibility and wearing comfort. Further validation via infrared thermal imaging (Fig. S35b) demonstrates that PASHE synchronizes effectively with skin temperature in real time, thereby preventing overheating during use and avoiding thermal discomfort upon removal. This intrinsic thermoresponsive behavior enhances user safety and comfort while minimizing the risk of heat-induced skin irritation. Capitalizing on its inherent flexibility, biocompatibility, and high ionic conductivity, PASHE was designed as a high-performance strain biosensor for real-time monitoring of human motion and physiological signals. The sensing performance of the PASHE-based sensor were systematically investigated, as illustrated in Fig. 6. Under varying applied strains (30%–200%, Fig. 6a) and testing speeds (50–300 mm min^−1^, Fig. 6b), the sensor exhibits highly stable and repeatable Δ*R*/*R*_0_ signals, affirming the reliability and reproducibility of its electromechanical characteristics. The sensitivity, represented by the gauge factor (GF), reaches a value of 1.1 within a strain range of 0–130% (Fig. 6c). Moreover, a strong linear correlation (*R*^2^ = 0.996) is observed between Δ*R*/*R*_0_ and strain, indicative of outstanding signal linearity. This performance is attributed to the highly reversible conductive pathways enabled by the hydrogel’s low hysteresis, which facilitates precise quantification of mechanical deformation via resistance monitoring. The response speed, a critical parameter for flexible electronics and data transmission efficiency, was assessed through five consecutive loading–unloading cycles. As depicted in Fig. 6d, the PASHE-based sensor demonstrates rapid response times of approximately 130 ms (loading) and 150 ms (unloading). The sensor also exhibits well-defined, stepwise resistance transitions under incremental strains (0–100%) during both loading and unloading (Fig. 6e), highlighting its broad sensing range and reliable cyclic performance. Moreover, owing to its excellent elasticity and minimal hysteresis, the mechanical stress and Δ*R*/*R*_0_ signal vary synchronously with strain (Fig. 6f), underscoring the sensor’s capability for real-time motion tracking. The combination of ultra-low hysteresis and high linearity ensures consistent Δ*R*/*R*_0_ output at any given strain, regardless of loading direction. The detection limit is further verified by applying a minimal strain of 0.5% (Fig. S36), which produces a discernible resistance shift, attesting to the sensor’s high sensitivity. Long-term operational stability was evaluated over 500 continuous loading–unloading cycles at 30% strain (Fig. 6g). The Δ*R*/*R*_0_ response remains highly stable throughout, demonstrating excellent mechanical robustness and sensing durability. In addition, the PASHE-based sensor was assembled into an electrode patch for electroencephalography (EEG) monitoring. This patch electrode achieves a signal-to-noise ratio (SNR) of 25.04 dB (Fig. S37), substantially exceeding the 18.42 dB obtained using the commercial gel electrode. These results affirm the superior signal fidelity and reliability of the PASHE-based sensor in diverse electrophysiological applications.

Leveraging the exceptional sensing performance of the PASHE-based sensor and PASHE’ inherent advantages in Zn-based energy storage systems, we developed a self-powered wireless sign language recognition platform. This system integrates the PASHE-based sensor with three serially connected Zn//I_2_ batteries (Fig. 6h). The PASHE-based sensor is conformally laminated onto the metacarpophalangeal joints, allowing real-time translation of articulatory gestures into digital communication signals via time-resistance responses. Signal encoding follows the International Morse Code, where alphabetic characters, numbers, and punctuation are represented by sequences of “dots” and “dashes”. Specifically, a rapid finger flexion-return motion generates a transient resistance change, defined as a “dot”, whereas a maintained flexion before return produces a sustained resistance shift, interpreted as a “dash”. Based on this principle, users can express their thoughts by flexing their fingers regularly to generate distinct resistance signals. According to the Morse code summary (Fig. S38), voice signals like “Help”, “Cold”, “Heat”, “Pain”, “Danger”, and “SOS” can be easily identified by resistance variations, thereby ensuring that the information is accurate (Fig. 6i–n).

Expanding to neurophysiological monitoring, the PASHE-based sensor demonstrates accurate bio-signal acquisition, including EEG and electrooculography (EOG). The signals are then wirelessly transmitted to the display terminal via the Bluetooth module, as illustrated in Fig. 6o. EEG signals acquired via the PASHE-based sensor demonstrate comparable amplitude fidelity and sensitivity to commercial-grade electrodes, while exhibiting superior signal curve stability under identical testing configurations and temporal durations (Fig. 6p). Figure. 6q illustrates the capability of the PASHE-based sensor in tracking ocular dynamics through periodic EOG waveforms. Time-resolved potential oscillations correlate precisely with variable blink rates (slow vs. rapid), with significant enhancement in signal frequency and repeatability during rapid blinking. These collective findings, embracing full cells and sensor systems, demonstrate exceptional promises of PASHE as hydrogel electrolyte and wearable biopotential sensor for high-performance batteries and real-time electrophysiological monitoring.

## Conclusions

In summary, we have develop an in situ fabricated polyhydroxy hydrogel electrolyte with tuned interface chemistry for ultra-stable biosensing-compatible ZBs. Comprehensive characterizations and simulations reveal that the hydroxyl-rich L-SBS component synergistically functions as Zn surface selective adsorbent, EDL & SEI modifiers, Zn(002)-facet deposition promoter, and H-bond disruptor. These integrated functions collectively suppress water-induced side reactions and enable planar Zn deposition, significantly improving the durability and reversibility of Zn plating/stripping. Consequently, the Zn//Zn cell using PASHE survives up to 3300 h at 0.5 mA cm^−2^/0.5 mAh cm^−2^, while the Zn//Cu cell harvests ultra-high average CE of 99.6% over 1200 cycles. More encouragingly, PASHE facilitates exceptional cyclability in full cells, that are Zn//I_2_ battery retain 94.9% capacity after 9000 cycles and ZHC preserves 98.0% capacity after 43,000 cycles. Crucially, an integrated PASHE-based biosensing system powered by cascaded Zn//I_2_ batteries successfully enables real-time healthcare monitoring. This in situ engineering strategy for polyhydroxy-functionalized hydrogel electrolytes paves the way for developing durable aqueous Zn-based energy storage systems and flexible biosensing electronics.

## Supplementary Information

Below is the link to the electronic supplementary material.

Supplementary file1 (DOCX 13337 KB)
