# Supplementary material for: Polyhydroxy Hydrogel Electrolyte with In Situ Tuned Interface Chemistry for Ultra-Stable Biosensing-Compatible Zinc Batteries
Source: Nanomicro Lett. 2026 Jan 26;18:217. doi: 10.1007/s40820-025-02061-z (PMC12832600; doi:10.1007/s40820-025-02061-z)
Supplement: Supplementary file 1 — Supplementary file1 (DOCX 13337 KB) [file 40820_2025_2061_MOESM1_ESM.docx]

Supplementary Information

**Polyhydroxy Hydrogel Electrolyte with In Situ Tuned Interface Chemistry for Ultra-Stable Biosensing-Compatible Zinc Batteries**

Fengjiao Guo^1^, Chunjiang Jin^1^, Hongyu Mi^1,^*, Ziqiang Liu^1^, Bo Xu^1^, Wenhan Jia^1^, Guozhao Fang^2,^*, Jieshan Qiu^3,^*

^1^ School of Chemical Engineering and Technology, Xinjiang University, Urumqi 830017, People’s Republic of China

^2^ School of Materials Science and Engineering, Key Laboratory of Electronic Packaging and Advanced Functional Materials of Hunan Province, Central South University, Changsha 410083, People’s Republic of China

^3^ State Key Laboratory of Chemical Resource Engineering, College of Chemical Engineering, Beijing 100029, People’s Republic of China

*Corresponding author. E-mail: mmihongyu@xju.edu.com (H. Mi), fg_zhao@csu.edu.cn (G. Fang), qiujs@mail.buct.edu.cn (J. Qiu)

**1. Experimental section**

**1.1 Chemical reagents and materials**

Acrylamide (AM), L-sorbose (L-SBS), zinc sulfate heptahydrate (ZnSO_4_·7H_2_O), *N,N*'-methylenebisacrylamide (MBAA), and ammonium persulfate (APS) were produced by Sigma-Aldrich Co., Ltd. Zinc sheets were provided by Qingyuan Metal Co., Ltd.

**1.2 Preparation of petroleum pitch-based activated carbon material**

In brief, 30 g petroleum pitch was added to a three-neck flask and stirred at 230 ℃ in an oil bath and air atmosphere for 12 h. After cooling to room temperature, the petroleum pitch was heated to 450 ℃with a heat rate of 5 ℃ min^−1^ in Ar atmosphere for 2 h. Afterward, the pre-carbonized material was mixed with KOH (the mass ratio of 1:3) and heated to 800 ℃ with a heat rate of 5 ℃ min^−1^ in Ar atmosphere for 2 h. Finally, the product was washed with 1 M HCl solution and deionized water and dried to obtain petroleum-based activated carbon material, denoted as AC.

**1.3 Material characterization**

The tensile test were carried out by attaching the hydrogel samples (20.0×10.0×1.5 mm) to two clips at 150 mm min^−1^ using a tensile machine (ZQ-990LB). The adhesion strength was examined by the lap-shear measurement. The surficial component evolution of Zn electrodes after cycling was analyzed by X-ray photoelectron spectroscopy (XPS, Thermo FisherScientific ESCALAB 250XI) coupled with Ar^+^ sputtering. The Fourier transform infrared (FTIR, VERTEX 70 RAMI), Raman (HR Evlution), and nuclear magnetic resonance (NMR, AVANCE NEO 600) spectroscopies were used to investigate the structure of hydrogel electrolytes. The tensile and compression tests were conducted by securing the hydrogel electrolytes to two clamps with a tensile testing machine (ZQ-990LB). The tensile tests were executed on specimens measuring 20.0×10.0×1.5 mm at a rate of 150 mm min^−1^. The compression tests were performed on cylinders with a diameter of 12 mm and a height of 25 mm. The morphology, structure, and component of cycled Zn foils were studied by scanning electron microscope (SEM, JEM-2100), X-ray diffraction (XRD, D8 advance), XPS (Thermo FisherScientific ESCALAB250Xi), and time of flight secondary ion mass spectrometry (TOF-SIMS 5 iontof). The surface texture of Zn electrodes cycled in hydrogel electrolytes was checked by 2D-wide angle XRD (Bruker D8 Advance). The surficial appearances of Zn electrodes cycled in hydrogel electrolytes were observe by confocal laser scanning microscopy (CLSM, Leica TCS SP8) and atomic force microscopy (AFM, Bruker Dimension Icon).

**1.4 Electrochemical performance**

Symmetric cells with Zn electrodes were assembled to evaluate ionic conductivity and Zn^2+^ transference number ($t_{\text{Zn}{}^{2+}}$) of hydrogel electrolytes. The electrochemical impedance spectroscopy (EIS) measurement was carried out from 10^−1^ to 10^5^ Hz. The ionic conductivity (*σ*) of hydrogel electrolytes was calculated according to the equation S1.

$\sigma=\frac{l}{R\cdot A}$ (S1)

where *l*, *R*, and *A* are the thickness, the resistance, and the area of hydrogel electrolytes, respectively.

The $t_{\text{Zn}{}^{2+}}$ of hydrogel electrolytes was evaluated by EIS before and after the potential static, and calculated by the equation S2.

$t_{\text{Zn}{}^{2+}}=\frac{I_{\text{s}}(\Delta V-I_{0}R_{0})}{I_{0}(\Delta V-I_{\text{s}}R_{\text{s}})}$ (S2)

where *I*_0_, *I*_s_, ∆*V*, *R*_0_, and *R*_s_ standed for the initial current, the steady state current, the applied polarization voltage, the initial resistance, and the steady state resistance, respectively.

Chronamperometry (CA) tests were conducted on Zn//Zn cells with different hydrogel electrolytes using an electrochemical workstation (760F). A minimum nucleation overpotential of −0.1 V was initially applied to the Zn/PASHE/Zn cell to initiate zinc nucleation and deposition. Subsequently, the CA curves of the symmetric cells were measured at constant potentials of −0.15, −0.2, and −0.25 V, respectively. The obtained current-time transients were normalized and analyzed based on the Scharifker–Hills model. The nucleation density (N) can be calculated using the following formulas:

$N=\frac{I_{\text{m}}^{2}}{0.41z^{2}F^{2}D_{i}^{2}c^{2}k_{i}}$ (S3)

$k_{i}=\left(\frac{8\pi cM}{\rho}\right)^{1/2}$ (S4)

$D_{i}=\frac{I_{\text{m}}^{2}t_{\text{m}}}{0.1629\left(zFc\right)^{2}}$ (S5)

where *M*, *ρ*, *c*, *D*, *z*, and *F* represent the molar mass (g mol^−1^), the density of the deposition (g cm^−3^), the ZnSO_4_ concentration in the electrolyte (mol cm^−3^), the diffusion coefficient of the electroactive species (cm^−2^ s^−1^), the number of electrons involved in the redox process, and the Faraday constant, respectively.

Galvanostatic intermittent titration technique (GITT) measurements were performed on Zn//Zn symmetrical cells employing different hydrogel electrolytes. The tests were conducted by first discharging the cell at a current density of 1 mA cm^−2^ for 1 min, followed by a rest step of 1 min. This discharge-rest procedure was repeated for 60 cycles. Subsequently, the cell was charged at the same current density of 1 mA cm^−2^ for 1 min, again followed by a rest step of 1 min, and this charge-rest sequence was also repeated for 60 cycles.

The in situ EIS data were analyzed using the “DRT tools” software.

The Zeta potentials of the solid surface particles were measured by dispersing zinc powder (0.05 g) in 10 mL liquids (pure water and 1 M L-SBS solution) using a Nanoparticle size and Zeta potential analyzer (Malvern Zetasizer Nano ZS90). The electric double layer (EDL) measurement was carried out in Zn//Zn cells in a voltage range of −15 to 15 mV under various scanning rates. The EDL capacitance (*C*) was calculated through the equation S6:

$C=\frac{i_{\text{c}}}{v}$ (S6)

where *i*_c_ and *v* refer to capacitive current and scan rate, respectively.

The Tafel test was carried out on a three-electrode system concluding a saturated calomel electrode (reference electrode) and two Zn sheets (working and counter electrodes). Linear sweep voltammetry (LSV) test was conducted in a three-electrode system that comprises of an Ag/AgCl electrode and two Zn sheets. In situ measurement of hydrogen evolution flux was tested in a three-electrode configuration consisting of an Ag/AgCl electrode and two Zn sheets with the assistance of gas chromatography-mass spectrometry (GC-MS, Shimadzu GC-2010 Pro). Electrochemical quartz crystal microbalance (EQCM, QCM 200) was used to monitor the mass change during the Zn plating/stripping, in which the gold-plated quartz crystal electrode attached by Zn foil, Pt electrode, and Ag/AgCl electrode were used as working electrode, counter electrode, and reference electrode, respectively. The EQCM test was carried by chronopotentiometry on a CHI760E electrochemical workstation at 10 mA cm^−2^. The mass change (∆*m*) of the Zn foil was calculated based on the equation S7 [1].

$\Delta f=-2f_{0}^{2}\left(\mu_{q}\rho_{q}\right)^{-\frac{1}{2}}\Delta m/A$ (S7)

where ∆*f*, *f*_0_, *μ*_q_, *ρ*_q_, and *A* are frequency change, the resonant frequency of the quartz crystal, shear modulus of quartz (2.947 × 10^11^ g cm^−1^ s^−2^), the density of quartz (2.648 g cm^−3^), and piezoelectrically active crystal area, respectively. In situ Raman accompanied by a laser Raman spectrometer (HR Evlution) was carried out on Zn//Cu cells.

The gauge factor (GF) was calculated by the following equation:

$GF=\frac{\left(R-R_{0}\right)/R_{0}}{\varepsilon}$ (S8)

where *R* and *R*_0_ are the resistance values of PASHE at stretching and original states, respectively, and *ε* is the applied strain.

The monitoring of electroencephalograph (EEG) and electrooculography (EOG) signals is mainly realized by a device composed of electrodes, self-powered sensors, and acquisition software. EEG signals were collected by installing two PASHE patches on the forehead of volunteers and a reference patch behind the ears; EOG signals were collected by applying PASHE patches on the upper and lower parts of the volunteers' orbit, and the reference electrode was placed in the same position. The signal quality was measured using the SNR, a high SNR indicating that the signal power was greater than the noise power, resulting in higher fidelity. It could be calculated as follows [2]:

$SNR=20\log10\frac{RMS_{\text{signal}}}{RMS_{\text{noise}}}=20\log10\frac{\sqrt{\sum_{i=1}^{N}V_{\text{signal}}^{2}}}{\sqrt{\sum_{i=1}^{N}V_{\text{noise}}^{2}}}$ (S9)

where *V*_signal_ and *V*_noise_ denote the voltage values of the signal and noise, respectively, and *N* is the total number of samples.

The experiments involving human subjects have been performed with the full informed consent of the volunteer. To assess the skin irritation potential of PASHE, we performed a comparative abrasion test on the backs of the volunteers' palms. Skin samples were collected before and after each test cycle, with exposure durations of 0.5, 1, and 3 h applied to the test Materials. Five participants were enrolled in the comfort assessment study. Each PASHE was affixed to the back of the palm, with instructions for the volunteer to remove the sample immediately upon experiencing any discomfort, while recording the precise duration of wear. Changes in skin temperature beneath the gel patch were monitored in real time using a thermal imaging camera (Fotric 618C-L29).

**1.5 Density functional theory (DFT) calculations**

The adsorption energy was performed by the Vienna ab initio simulation package (VASP) using the Perdew-Burke-Ernzerhof of DFT and the projector augmented wave method. The adsorption energy (∆*E*_ads_) between the adsorbate and the Zn surface substrate was calculated using the equation S10:

∆*E*_ads_ = *E*_adsorbate@substrate_ – *E*_substrate_ – *E*_adsorbate_ (S10)

where *E*_adsorbate@substrate_ and *E*_substrate_ represent the total energies of the Zn surface with and without the adsorption of adsorbate, respectively. *E*_adsorbate_ is the total energy of the adsorbate.

The migration barrier along the migration paths was located using the climbing image nudged elastic band method with nine intermediate images along the pathway between the initial and the final states. Each identified transition state was further confirmed by the vibrational frequency analysis.

Molecular Dynamics (MD) simulations were conducted to investigate the Zn^2+^ solvation structure in the electrolytes as well as the interfacial structure between zinc electrodes and two distinct electrolyte systems. All simulations were performed using the Gromacs software suite. The number density distributions of key electrolyte components and the density distribution of hydrogen bonds were calculated as a function of distance from the zinc electrode surface.

**2. Supplementary figures and tables**


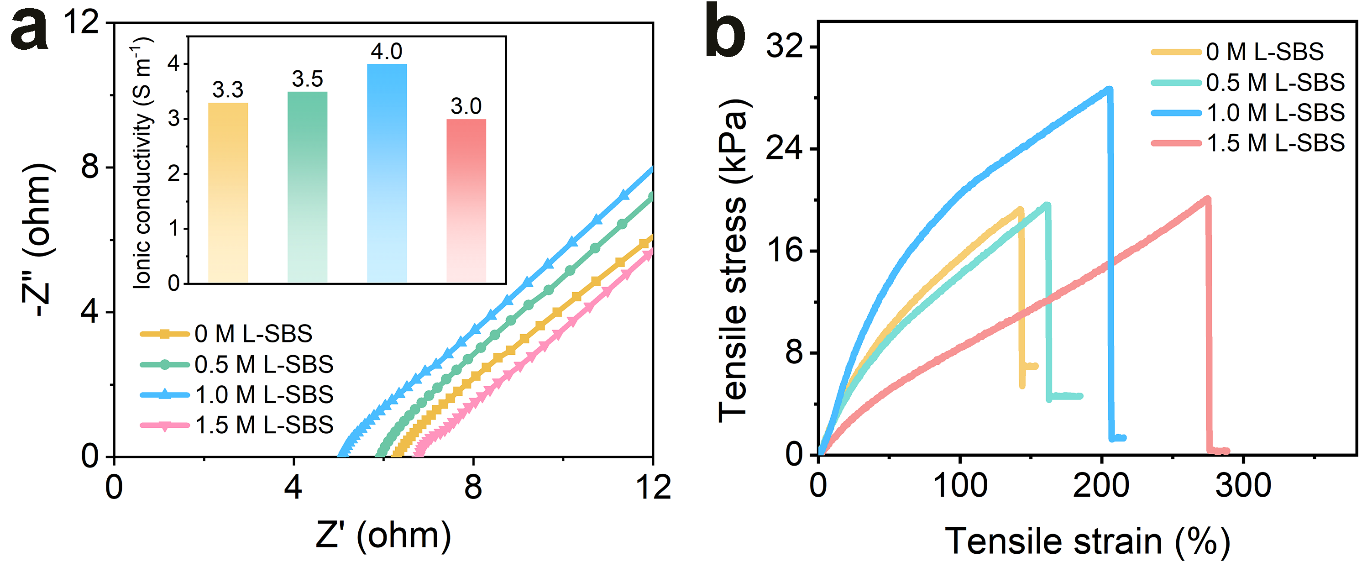


**Fig. S1. a** EIS plots and **b** tensile stress-strain curves of hydrogel electrolytes with 0, 0.5, 1.0, and 1.5 M L-SBS.


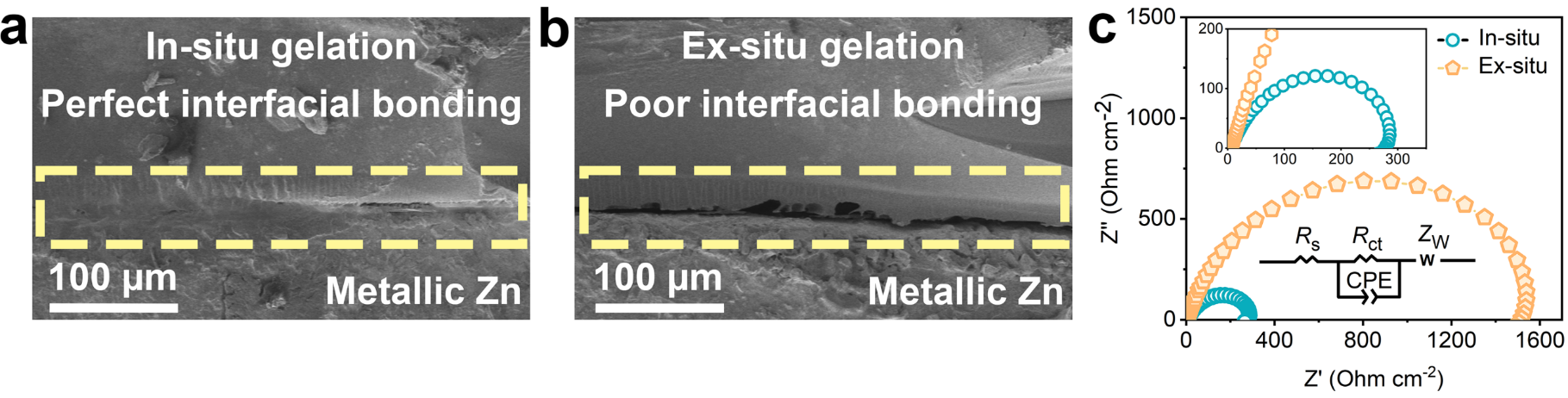


**Fig. S2.** SEM images of **a** in situ and **b** ex situ formed Zn-PASHE interfaces. **c** EIS plots of Zn//Zn cells using ex situ and in situ formed PASHE.


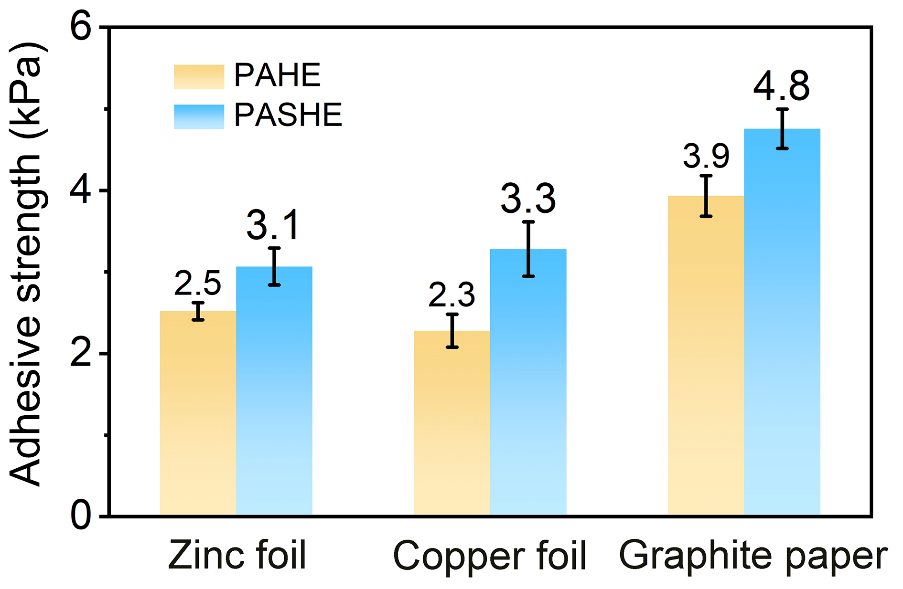


**Fig. S3.** Adhesive strengths of PAHE and PASHE to zinc foil, copper foil, and graphite paper.


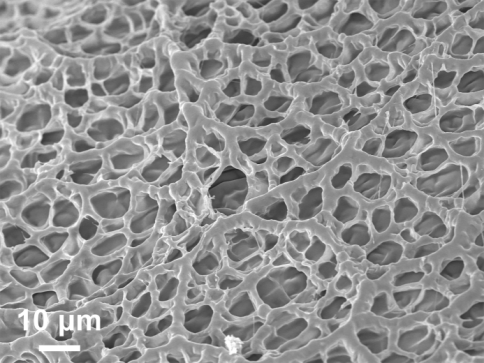


**Fig. S4.** SEM image of PASHE.


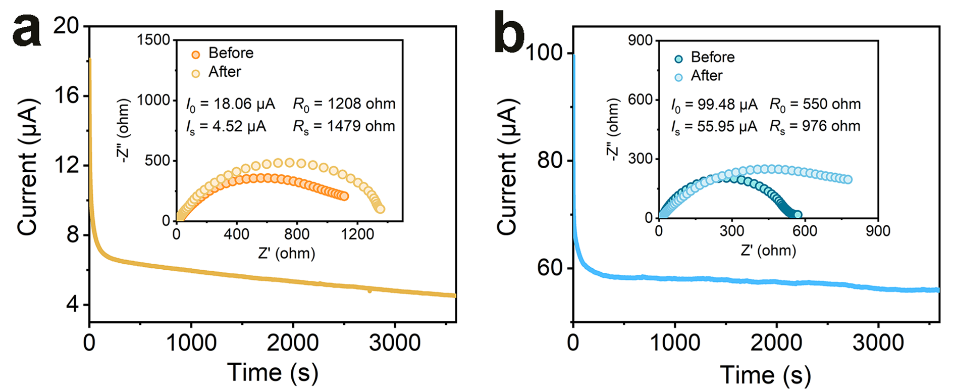


**Fig. S5.** *I*-*t* curves of Zn//Zn cells with **a** PAHE and **b** PASHE at an applied voltage of 10 mV (inset: EIS plots before and after polarization).


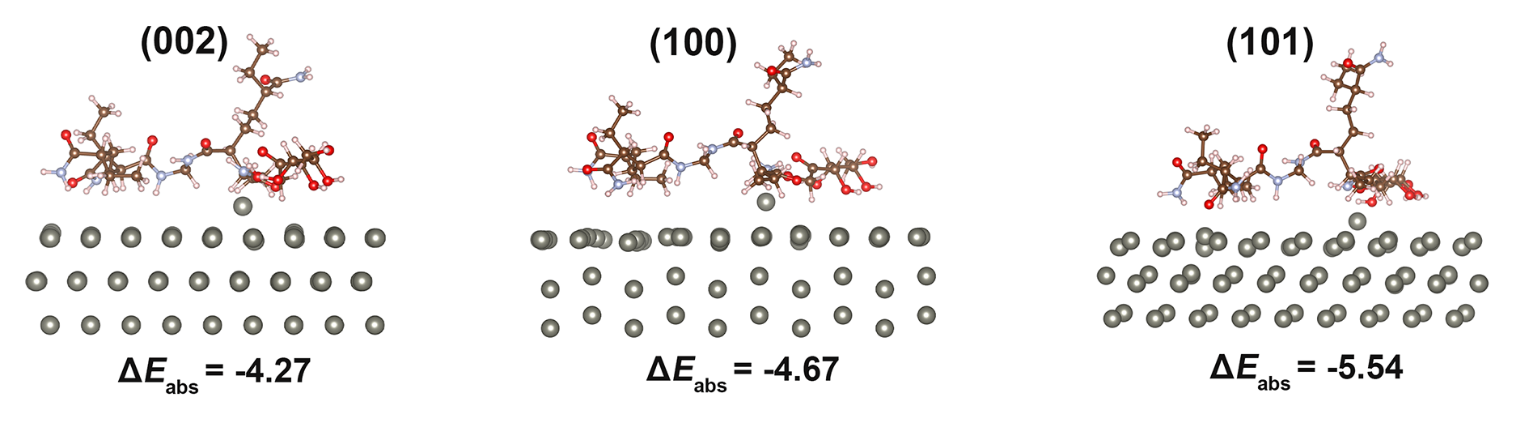


**Fig. S6.** Adsorption models and corresponding adsorption energies of Zn^2+^ on the PASHE-Zn interface.


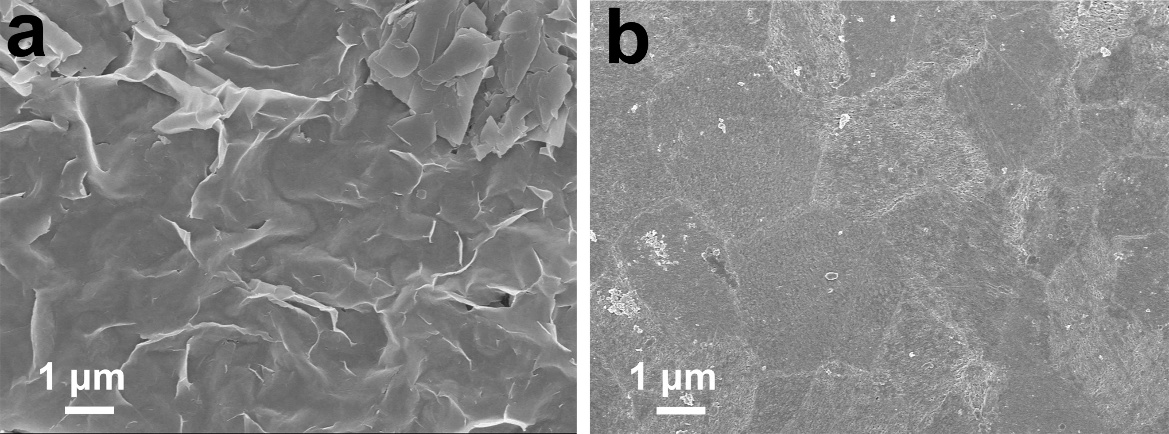


**Fig. S7.** SEM images of Zn deposits for Zn//Zn cells using **a** PAHE and **b** PASHE after 100 cycles at 5 mA cm^–2^.


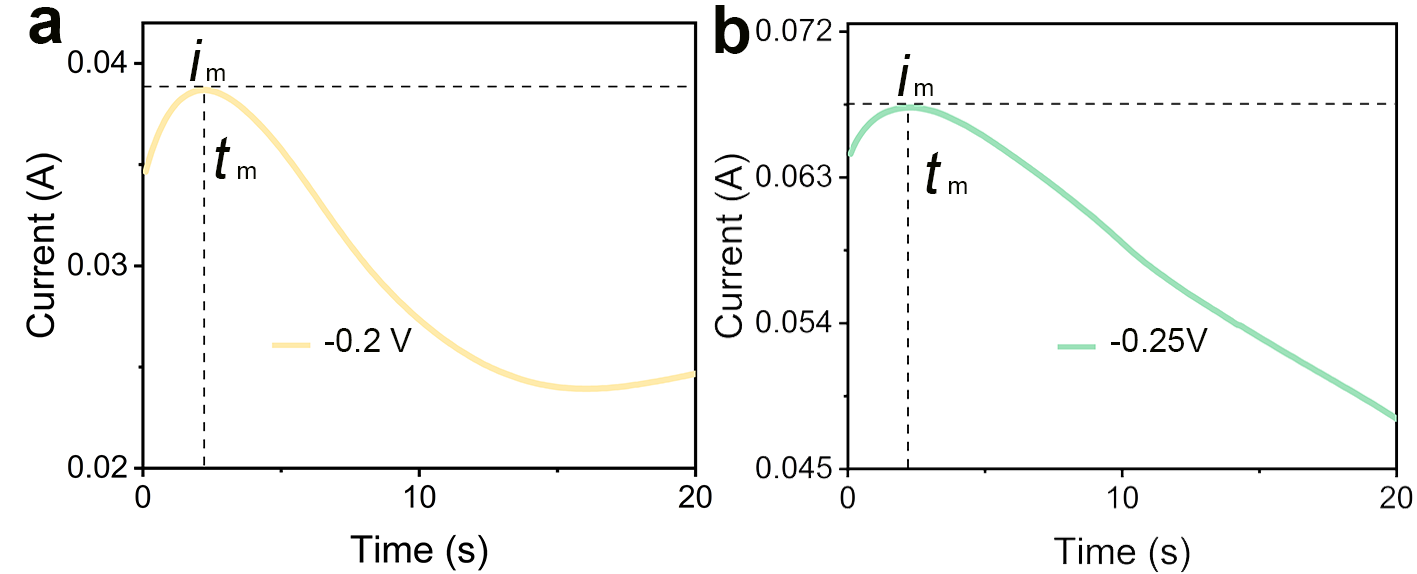


**Fig. S8.** Current-time transients obtained at a given potential in PAHE.


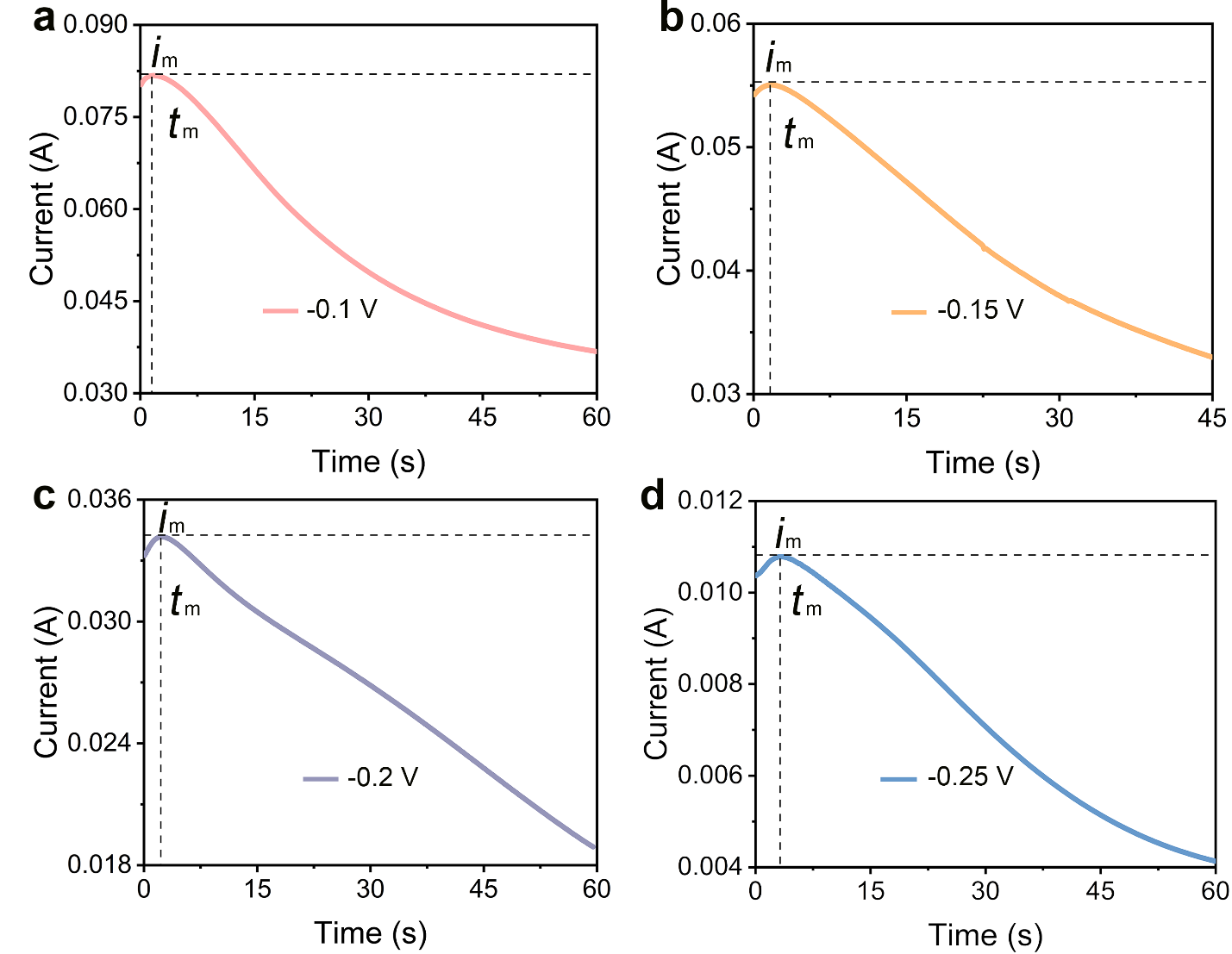


**Fig. S9.** Current-time transients obtained at a given potential in PASHE.


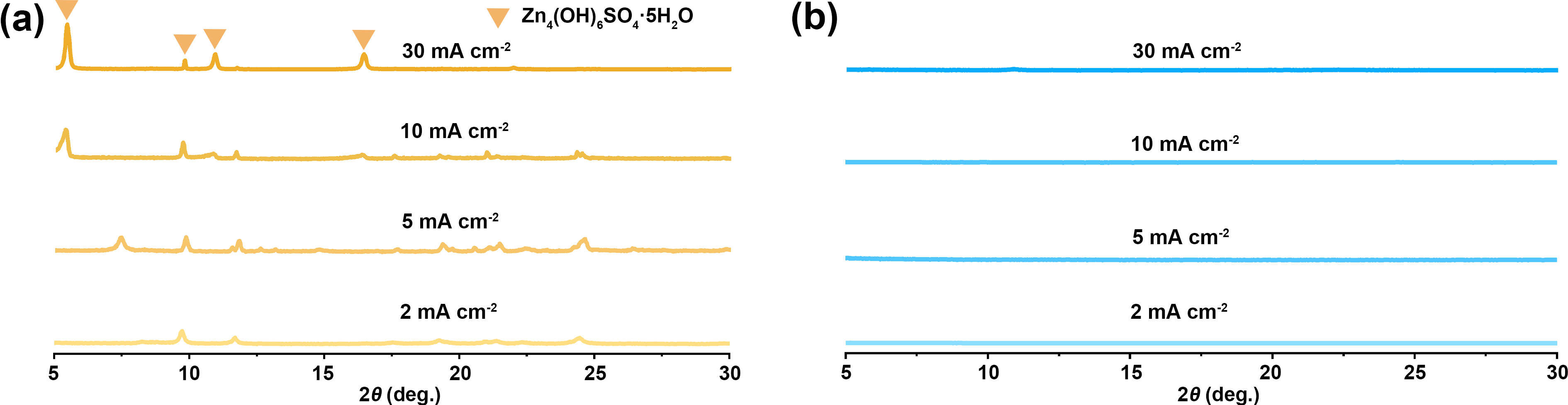


**Fig. S10.** XRD patterns of Zn electrodeposits in **a** PAHE and **b** PASHE.


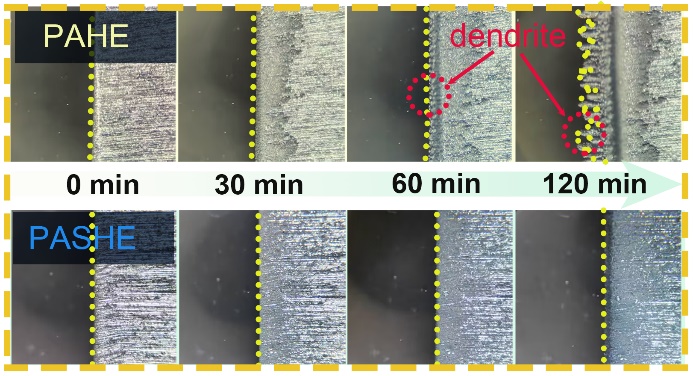


**Fig. S11.** Optical microscope images of Zn deposition in PAHE and PASHE at 1 mA cm^–2^.


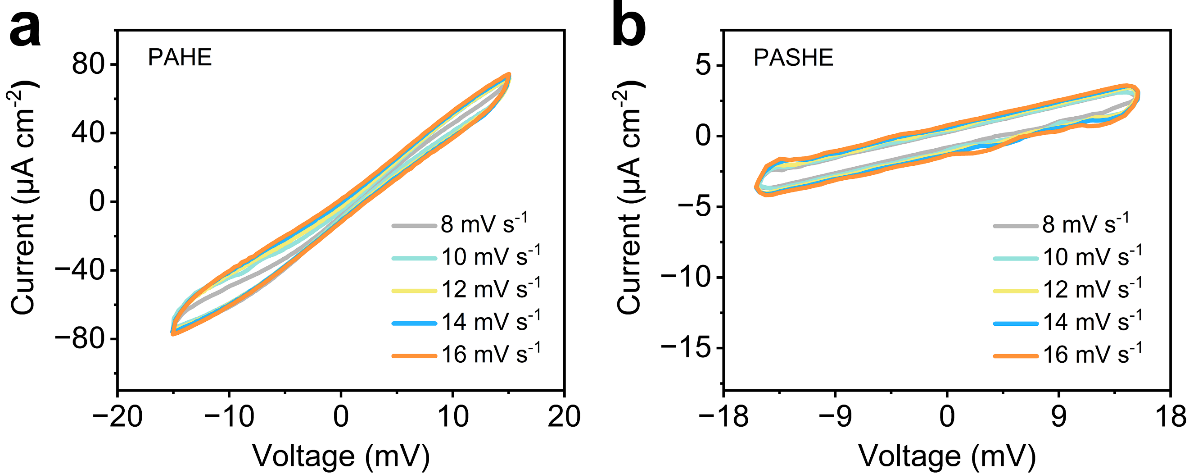


**Fig. S12.** CV curves of Zn//Zn cells with **a** PAHE and **b** PASHE.


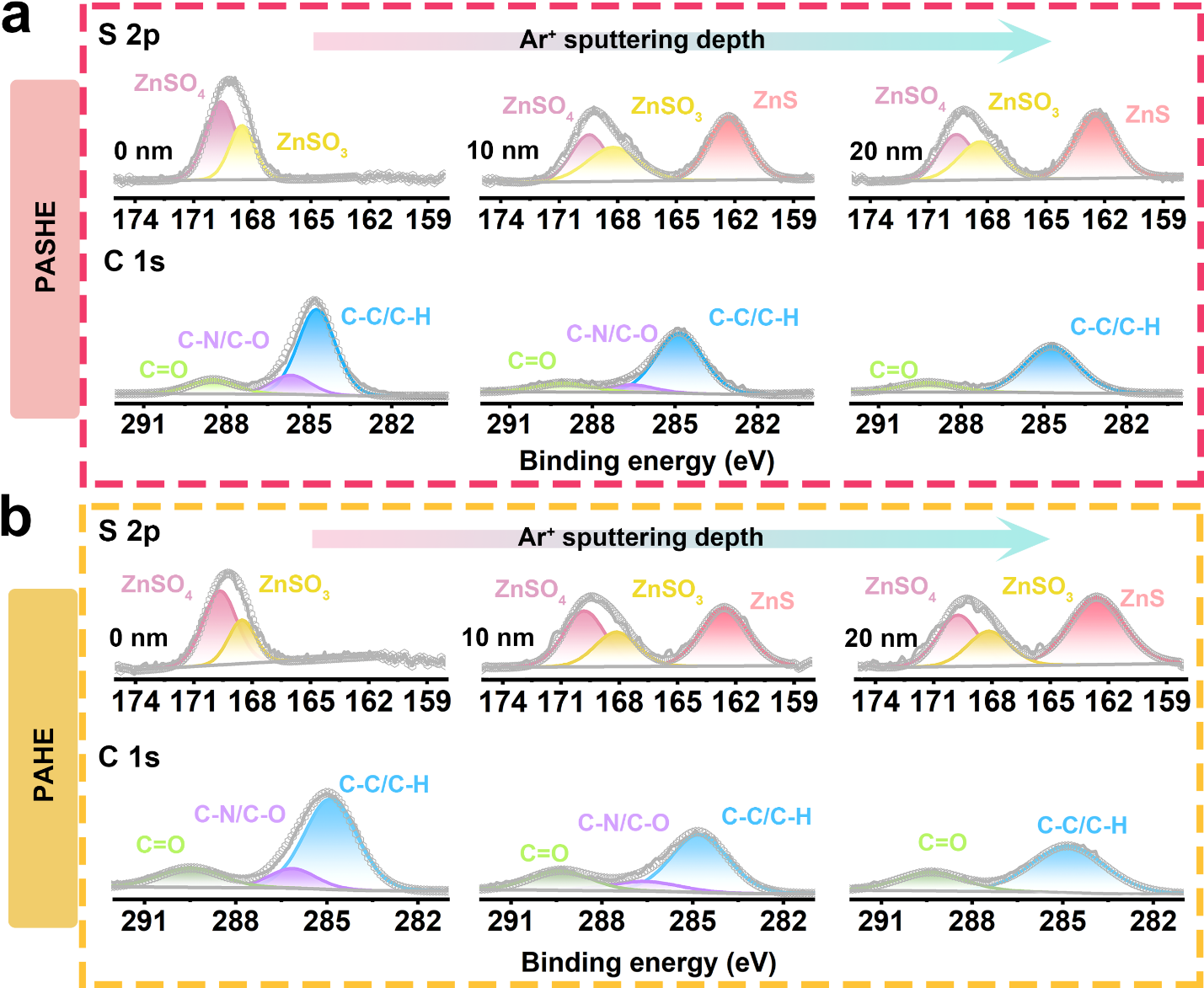


**Fig. S13.** High-resolution XPS depth spectra of Zn electrodes cycled in **a** PASHE and **b** PAHE at 5 mA cm^−2^/5 mAh cm^−2^ after 100 cycles.


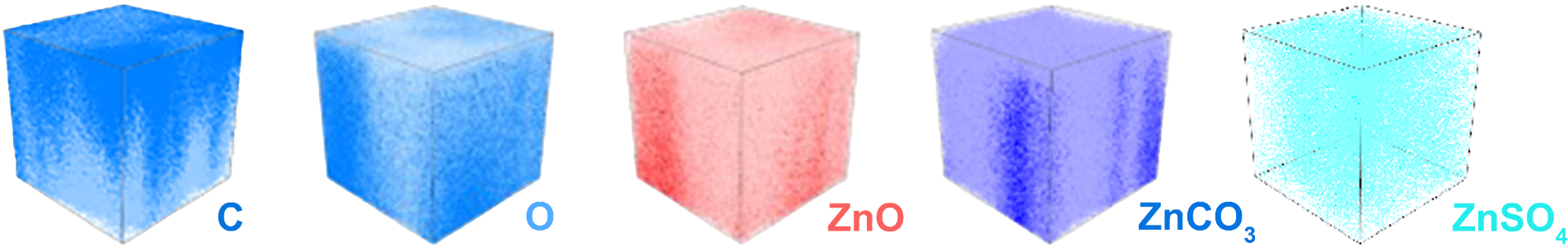


**Fig. S14.** Three-dimensional spatial distribution of elements and compounds for the Zn electrode cycled in PASHE.


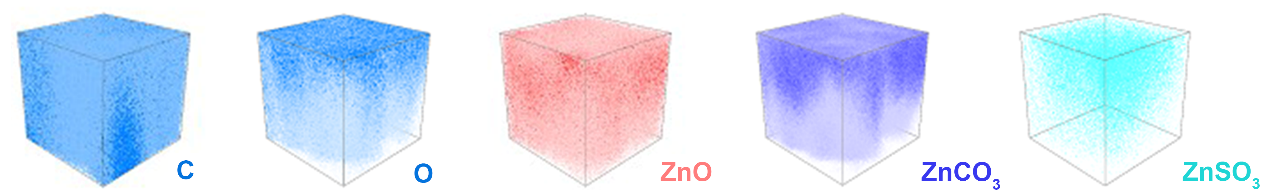


**Fig. S15.** Three-dimensional spatial distribution of elements and compounds for the Zn electrode cycled in PAHE.


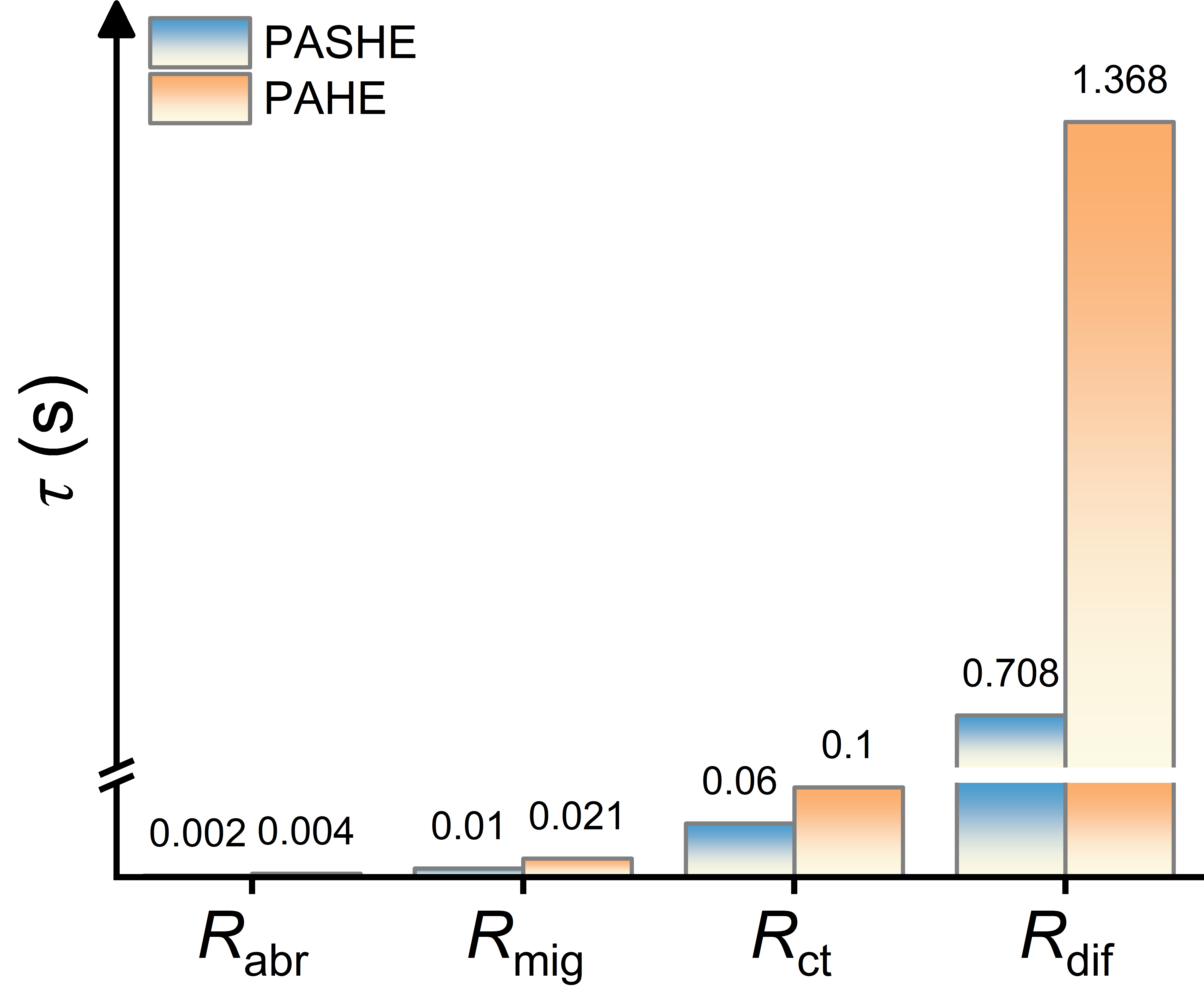


**Fig. S16.** The comparison of *τ* for diverse interfacial processes in PASHE and PAHE.


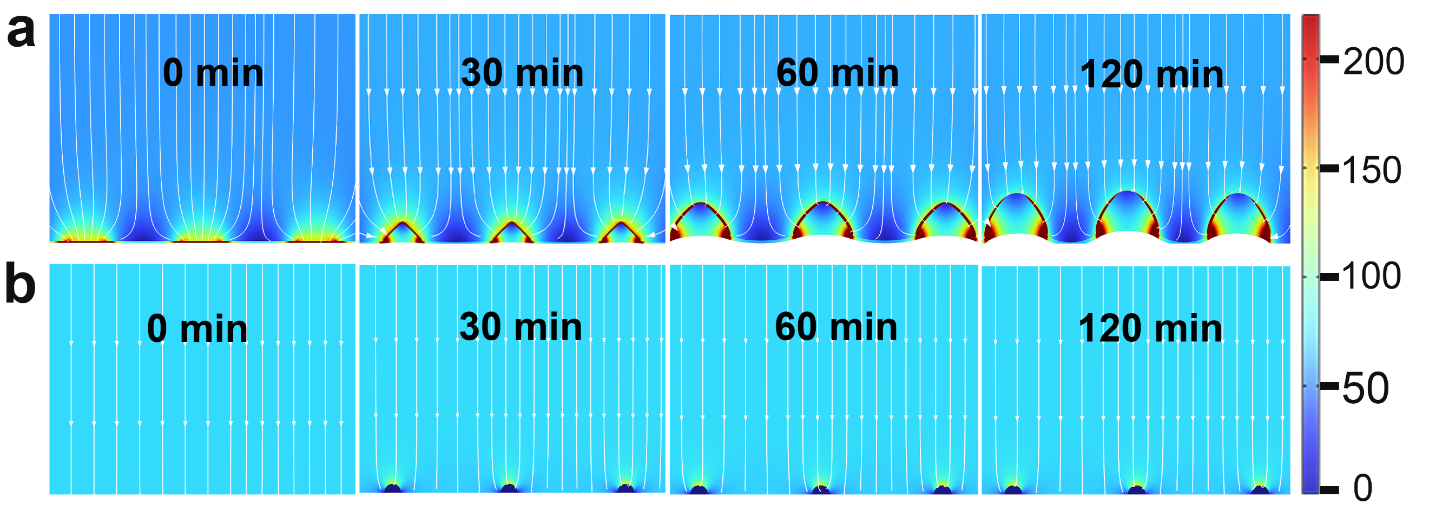


**Fig. S17.** Finite element modelling of electric field distribution and deposition morphology evolution in **a** PAHE and **b** PASHE during electrodeposition.


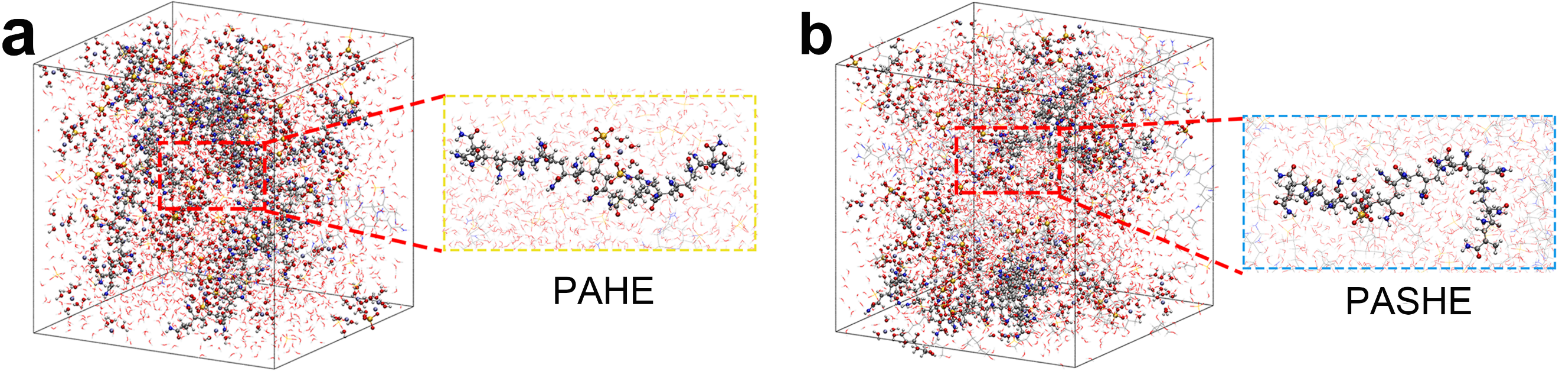


**Fig. S18.** MD simulation snapshots of **a** PAHE and **b** PASHE.


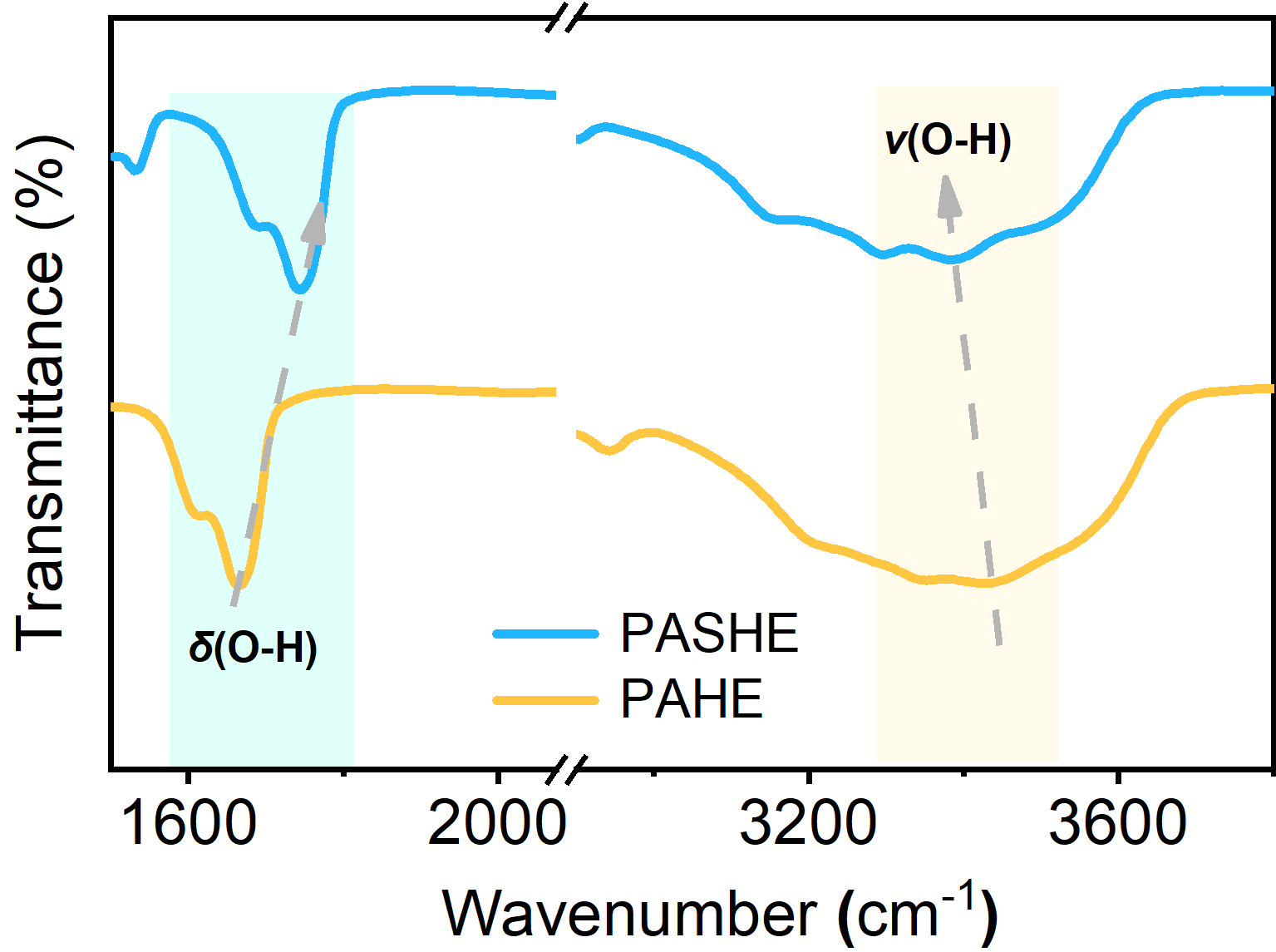


**Fig. S19.** FTIR spectra of PAHE and PASHE.

**
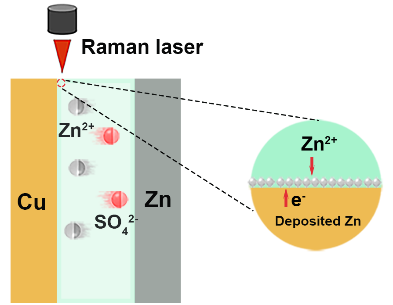
**

**Fig. S20.** Schematic diagram of in-situ Raman measurement.


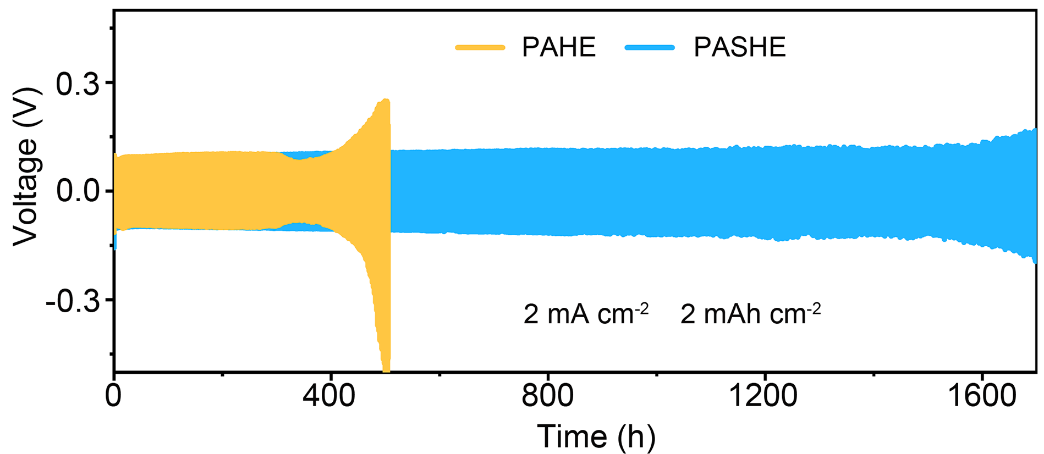


**Fig. S21.** Cycling performance of Zn//Zn cells using PAHE and PASHE at 2 mA cm^–2^/2 mAh cm^–2^.


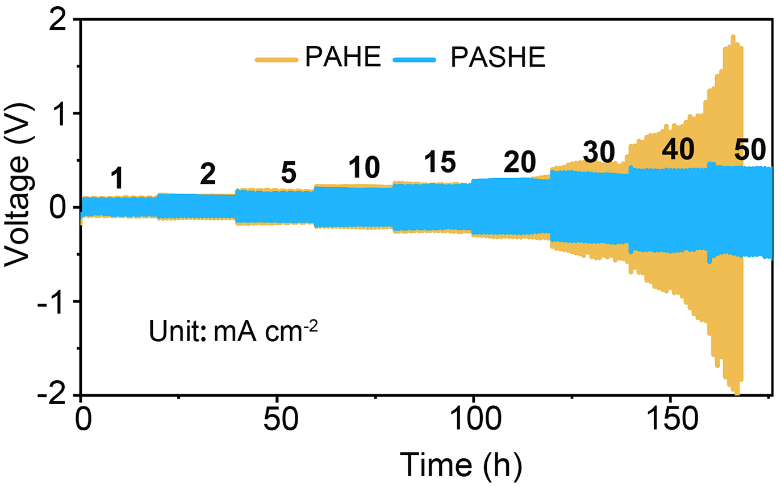


**Fig. S22.** Rate performance of Zn//Zn cells using PAHE and PASHE.


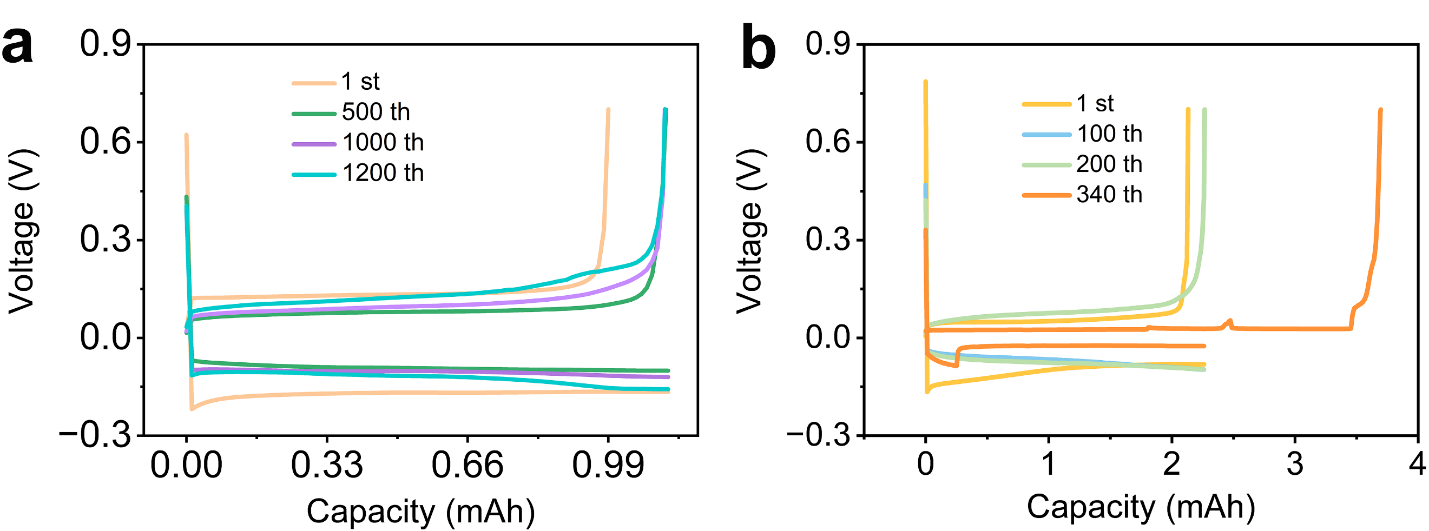


**Fig. S23.** The selected voltage profiles of the Zn//Cu cells using **a** PASHE and **b** PAHE.


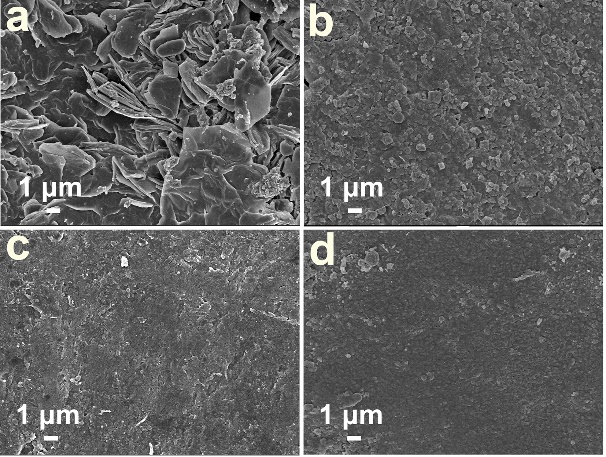


**Fig. S24.** SEM images of **a** Zn electrode and **b** Cu electrode after cycling in PAHE. SEM images of **c** Zn electrode and **d** Cu electrode after cycling in PASHE.


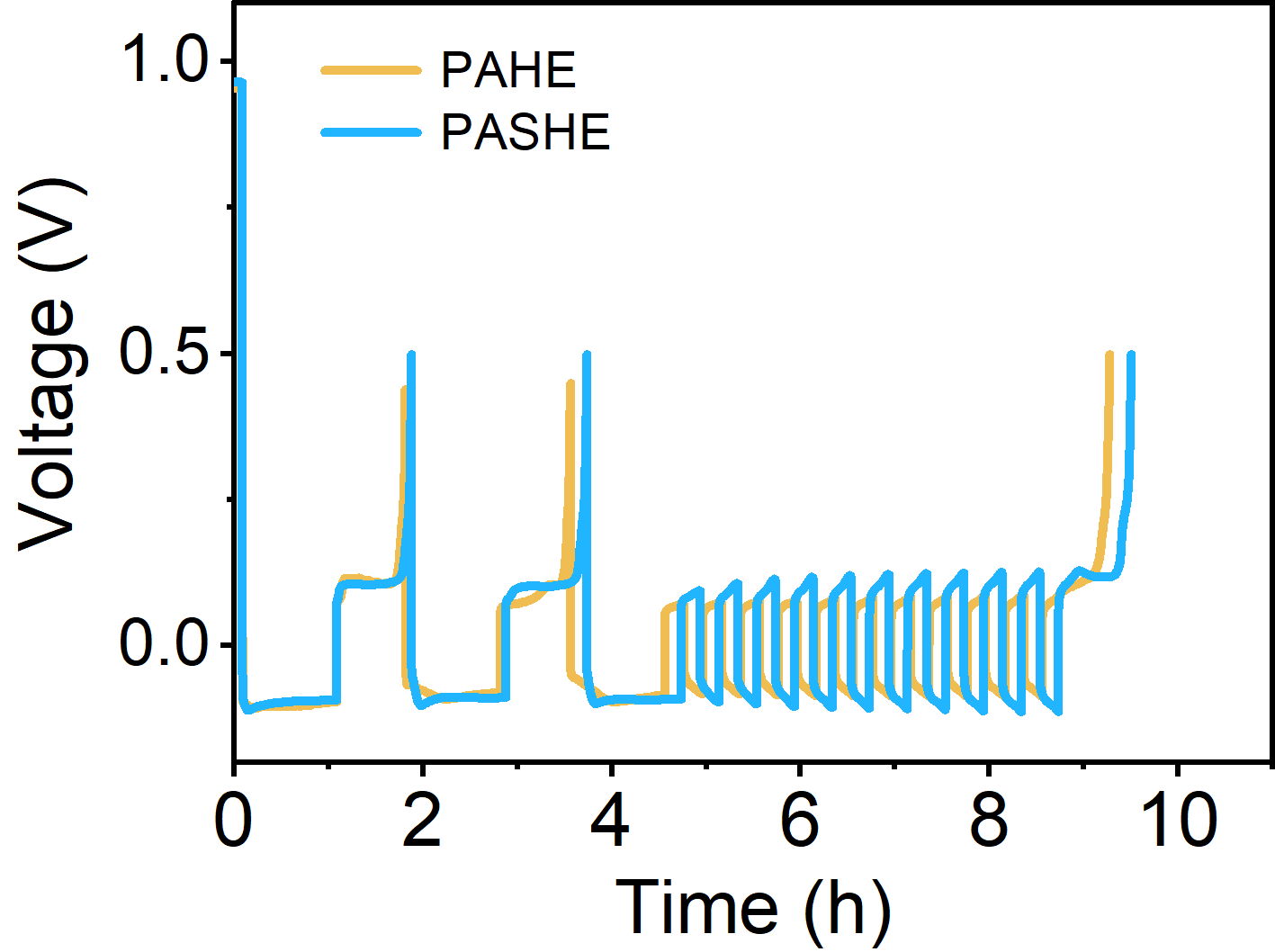


**Fig. S25.** Voltage profiles of Zn//Cu cells with PAHE and PASHE under the ‘reservoir half-cell’ protocol.


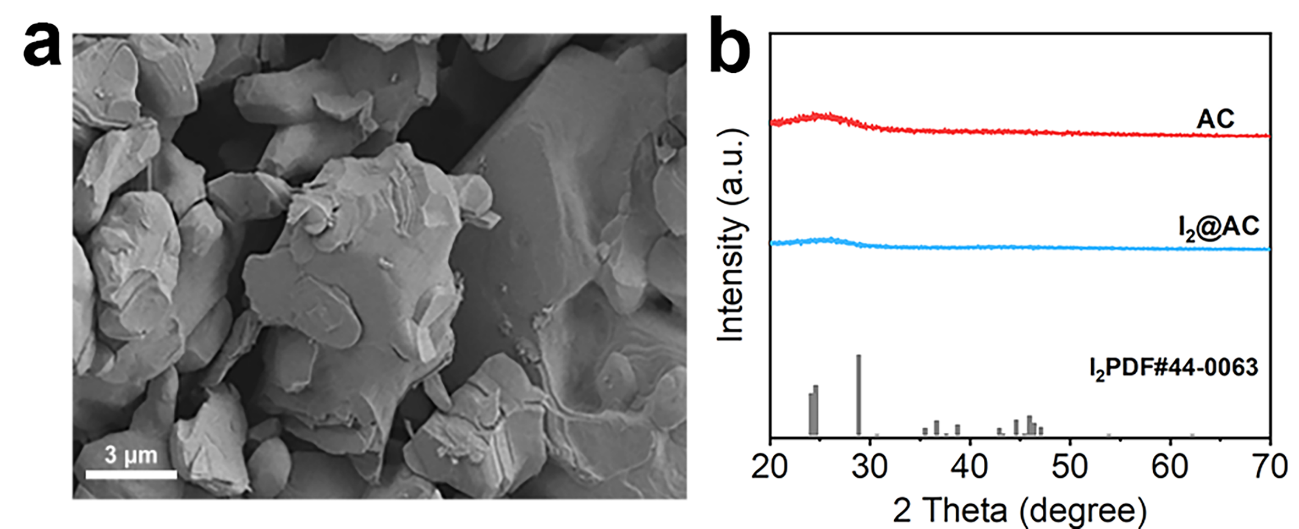


**Fig. S26. a** SEM image of I_2_ cathode. **b** XRD patterns of AC and I_2_ cathode.


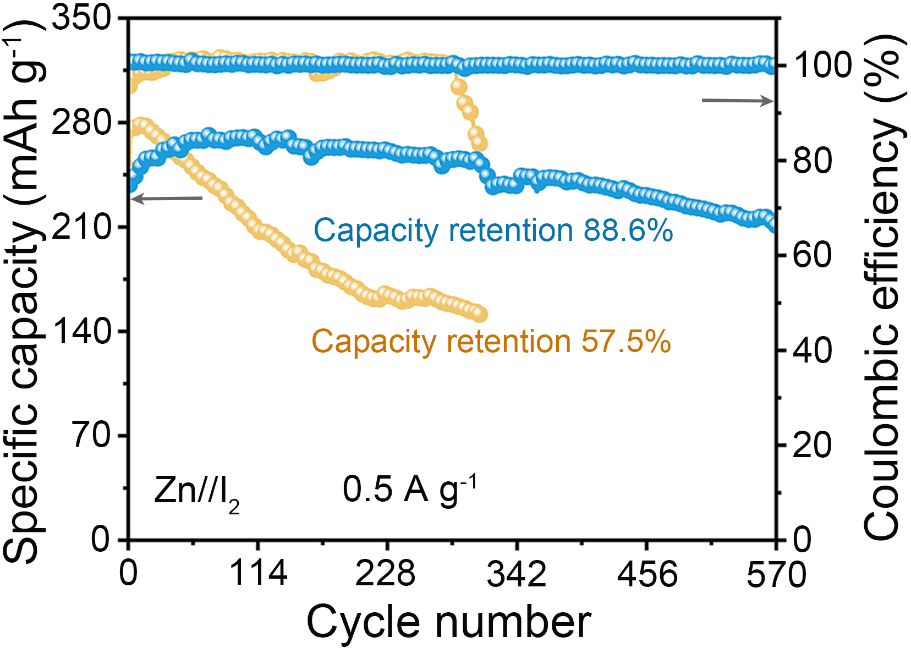


**Fig. 27.** Cycling stability of Zn//I_2_ batteries using PASHE and PAHE at 0.5 mA g^–1^.


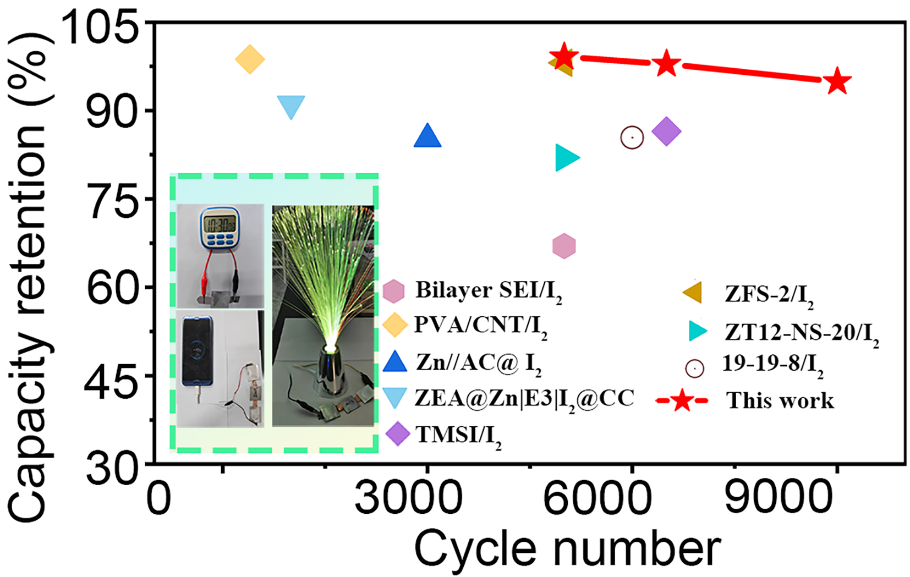


**Fig. S28.** Comparison of capacity retention of the PASHE-equipped Zn//I_2_ battery with recently reported batteries. Inset: Photographs of small appliances powered by PASHE-equipped Zn//I_2_ batteries.


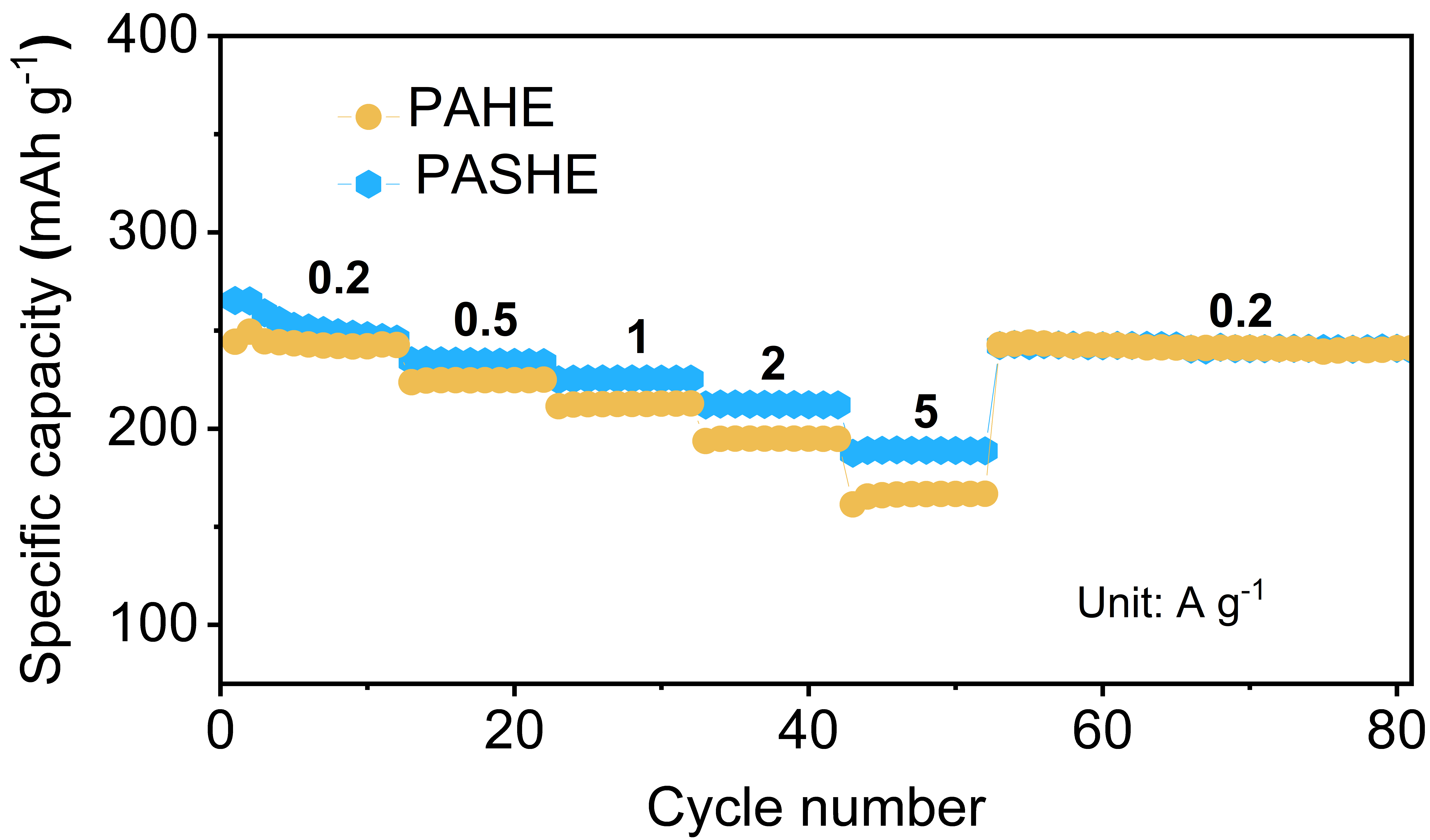


**Fig. S29.** Rate capability of Zn//I_2_ batteries with PASHE and PAHE.


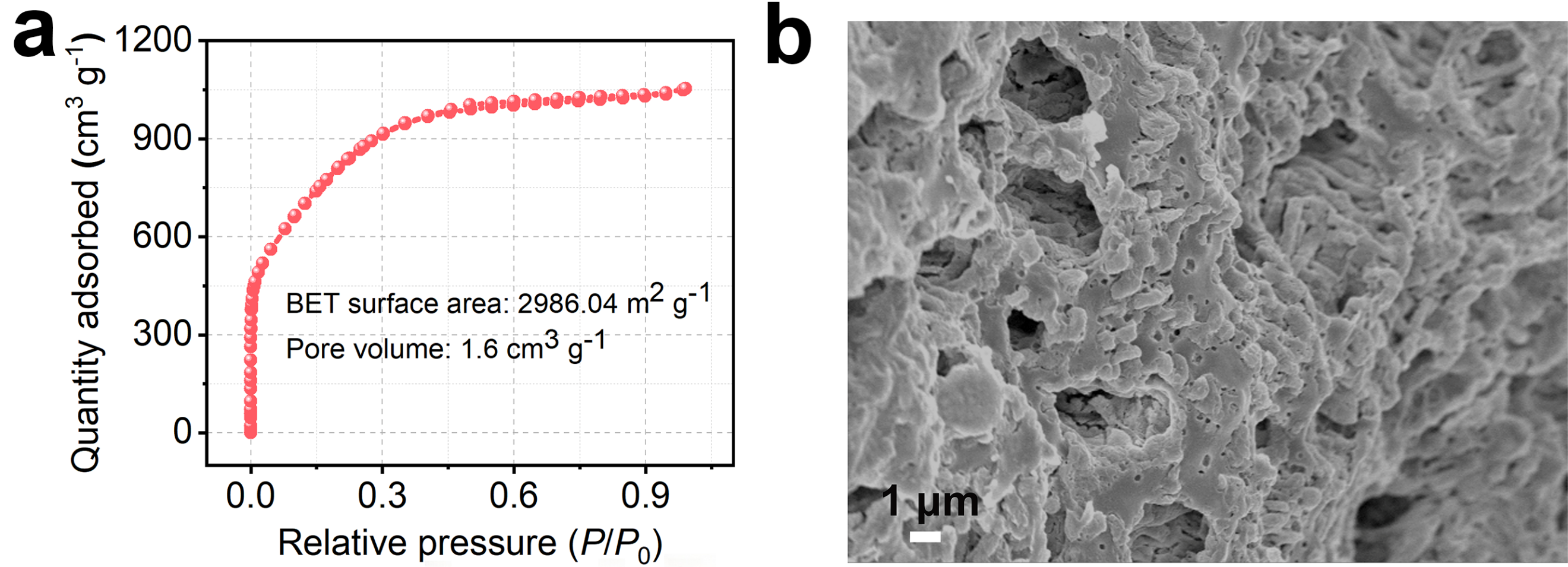


**Fig. S30.** **a** N_2_ absorption/desorption curves and **b** SEM image of AC.


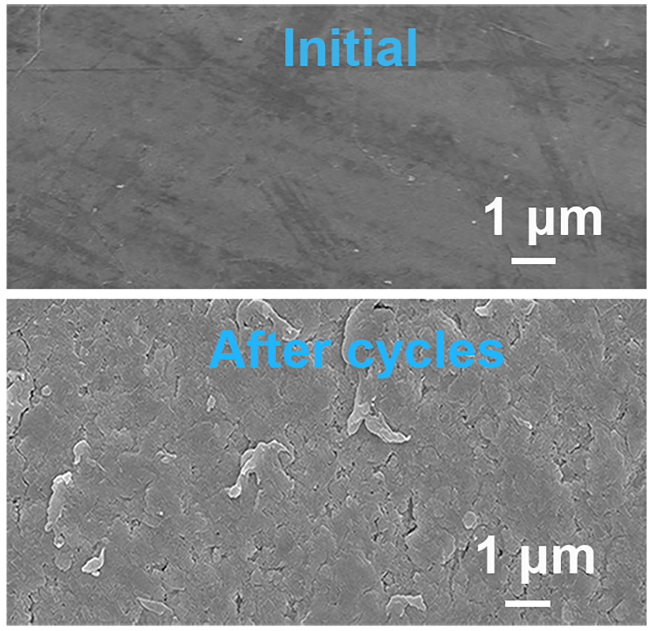


**Fig. S31.** SEM images of Zn anode in PASHE before and after cycling.


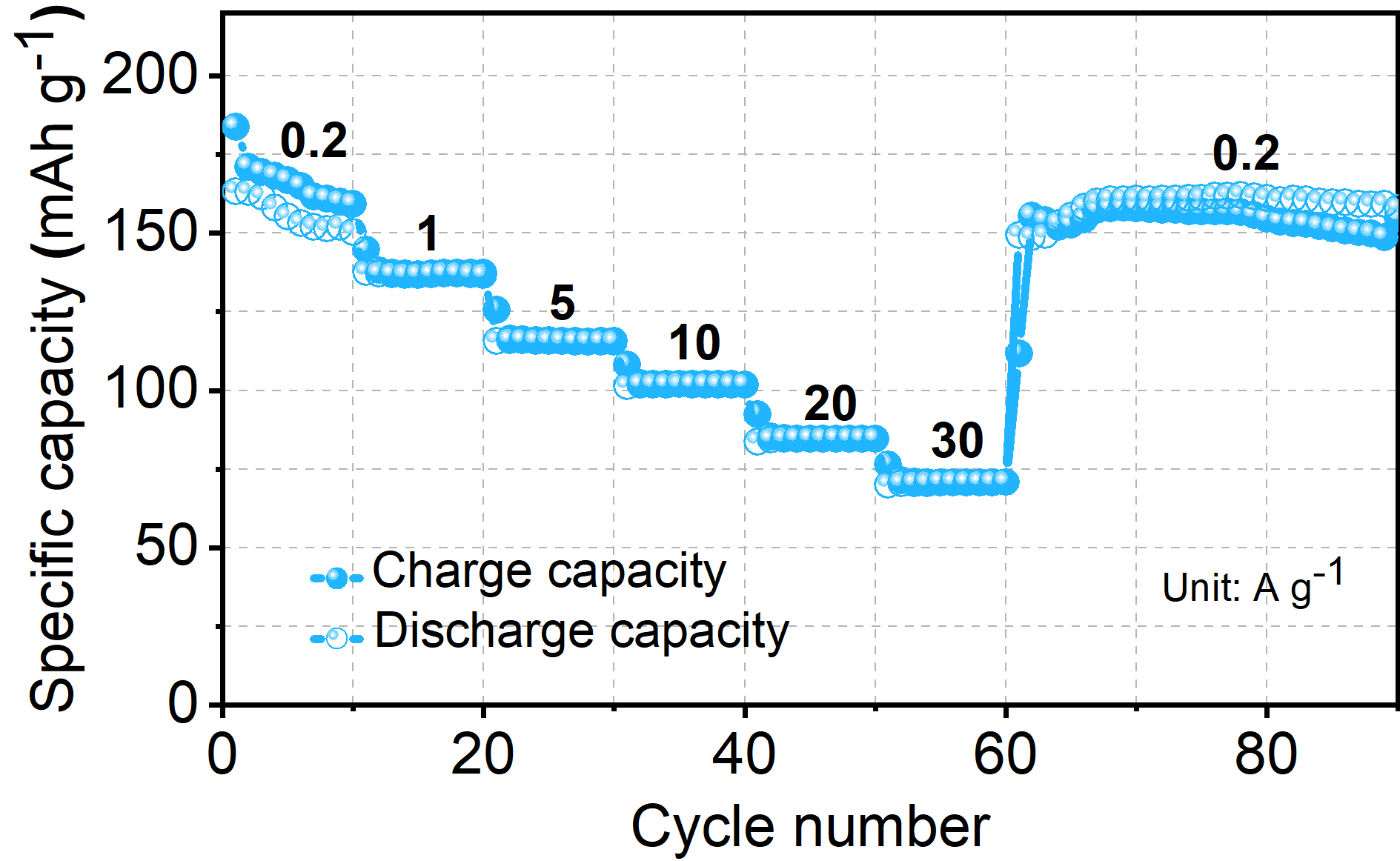


**Fig. S32.** Rate capability of the ZHC with PASHE.


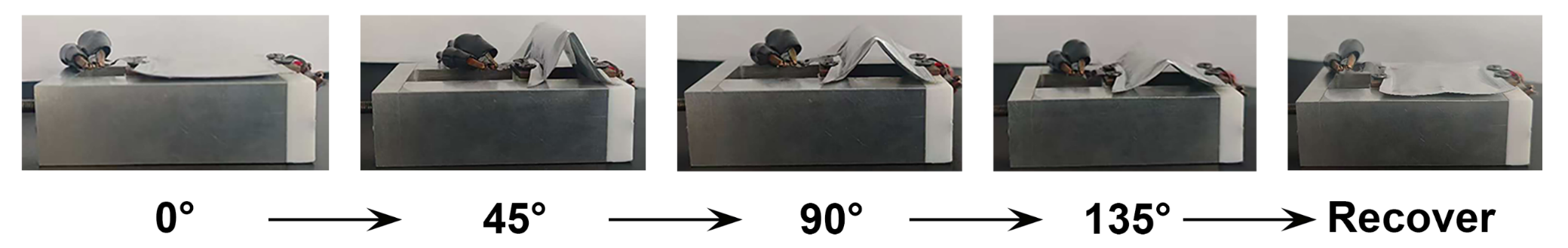


**Fig. S33.** Photos of the PASHE-equipped Zn//I_2_ battery under different bending deformations.


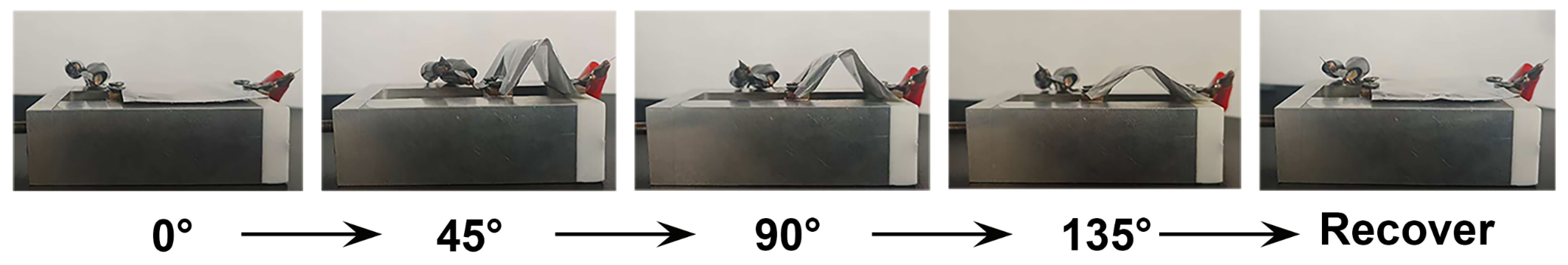


**Fig. S34.** Photos of the PASHE-equipped ZHC under different bending deformations.


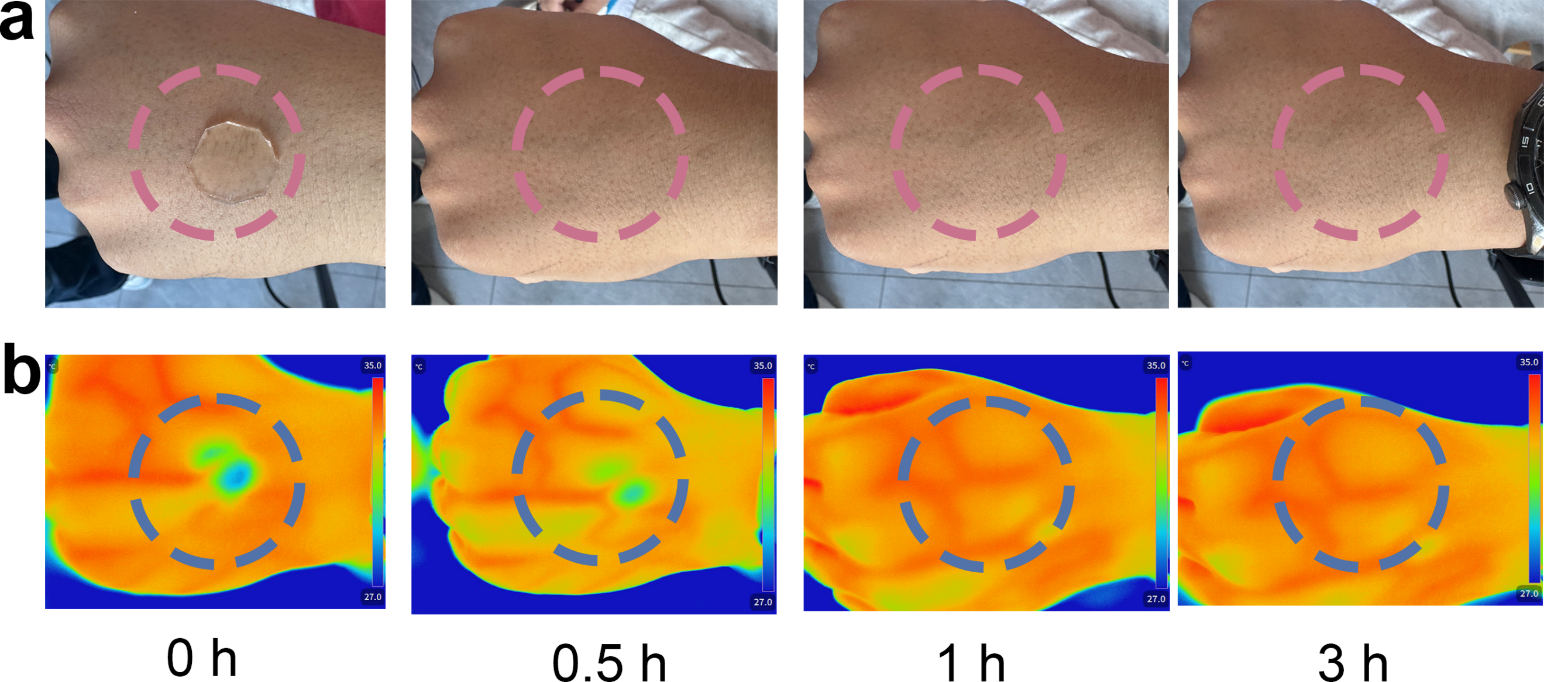


**Fig. S35.** **a** The digital image displays the inflammatory state of the skin on the left palm back covered with PASHE at different times. **b** Infrared images of the skin after adhesion of the PASHE for different times.


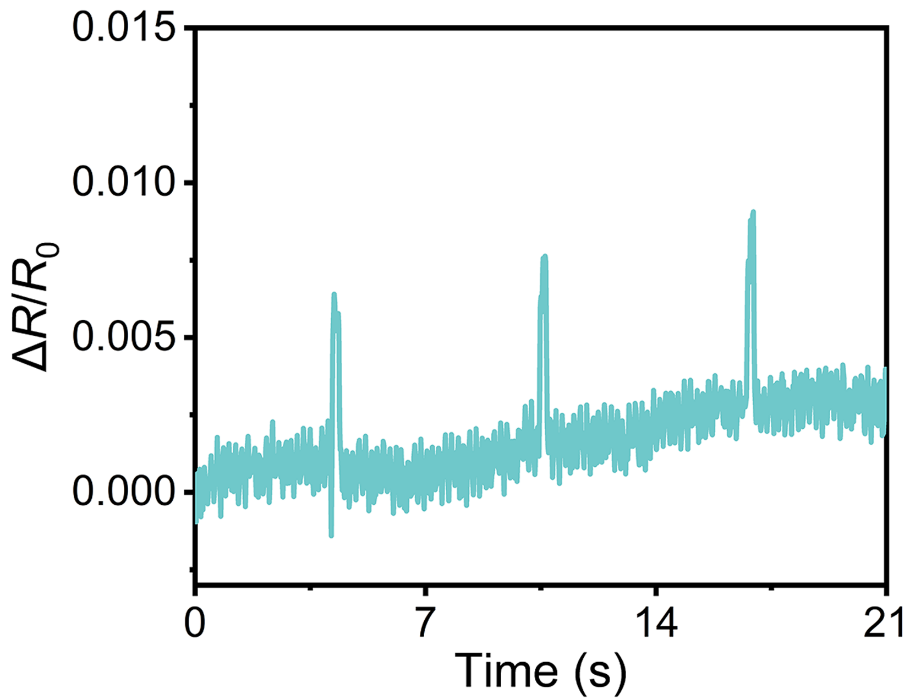


**Fig. S36.** Relative resistance curve of PASHE under 0.5% cyclic tensile strain.


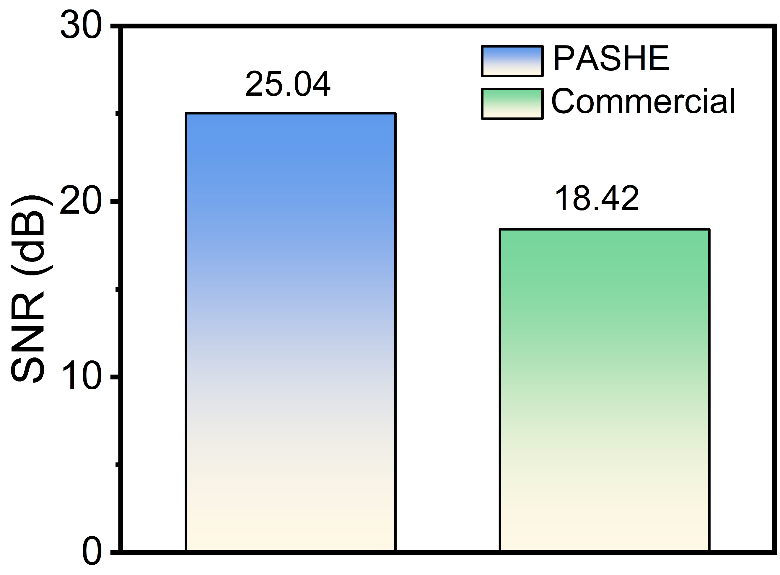


**Fig. S37.** SNR of EEG signals collected by PASHE and commercial hydrogel patch.


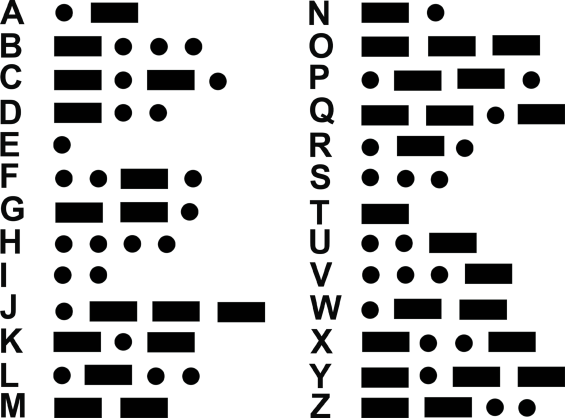


**Fig. S38.** The corresponding symbols of Morse code in the alphabet, numbers, and symbols table.

**Table S1.** Comparison of cycle duration between our PASHE-optimized Zn//Zn cell and reported cells.

| **Electrode** | | **Electrolyte** | | **Current density, capacity**  **(mA cm^-2^, mAh cm^-2^)** | | **Lifespan**  **(h)** | | **Ref.** |  |
| --- | --- | --- | --- | --- | --- | --- | --- | --- | --- |
| Zn | | 2 M ZnSO_4_ | | 0.5, 0.5 | | 500 | | [3] |  |
| P−Zn_40_ | | ZnSO_4_ | | 0.5, 0.5 | | 1000 | | [4] | |
| Zn@Bi/BiOCl | | Zn(Otf)_2_ | | 0.2, 0.2 | | 1200 | | [5] | |
| HMNVP/C@Zn | | Zn(CF_3_SO_3_)_2_ | | 0.2, 0.2 | | 1208 | | [6] | |
| ZnF_2_(90)/Zn | | | 1 M ZnSO_4_ | | 0.5, 1 | | 1500 | | [7] |
| Zn | | 50-DMSO+0.1 H_2_SO_4_ | | 0.5, 0.5 | | 1500 | | [8] |  |
| Zn | | HEE | | 0.5, 0.5 | | 1900 | | [9] |  |
| BC@Zn | | 1M ZnSO_4_ | | 0.5, 0.5 | | 2000 | | [10] |  |
| Zn | | HMEs | | 0.5, 0.5 | | 2150 | | [11] | |
| Zn | | ZnBF-VC. | | 0.5, 0.25 | | 2200 | | [12] | |
| MUA@Zn | | ZnSO_4_ | | 0.5, 0.5 | | 2400 | | [13] | |
| Zn | | N phase | | 0.2, 0.2 | | 2500 | | [14] |  |
| Zn | | P-4 | | 0.5, 0.5 | | 2500 | | [15] |  |
| Zn | | Zn-In electrolytes | | 0.5, 0.25 | | 2500 | | [16] |  |
| Zn | | ZS-In/CTS electrolytes | | 0.5, 0.5 | | 2500 | | [17] |  |
| COF-Zn | | 2M ZnSO_4_ | | 0.2, 0.2 | | 2900 | | [18] |  |
| Zn | | GPE | | 0.5, 0.5 | | 3000 | | [19] |  |
| **Zn foil** | | **PASHE** | | **0.5, 0.5**  **2, 2** | | **3300**  **1700** | | **This Work** |  |

**Table S2.** Comparison of cycle life between our PASHE-equipped Zn//I_2_ cell and reported cells.

| **Zn//I_2_ battery** | **Cycle** | **Capacity**  **retention**  **(%)** | **Ref.** |
| --- | --- | --- | --- |
| Zn//3-01-90-I_2_ with Zn(CF_3_SO_3_)_2_ | 500 (0.2 A g^−1^) | 99.3 | [20] |
| Zn//N2221-I_2_ with ZnSO_4_ | 4000 (0.2 A g^−1^) | 99.5 | [21] |
| Zn//GC-PAN-I_2_ with ZnSO_4_ | 2000 (20 C) | 98.71 | [22] |
| Zn//AC-I_2_ with ZnSO_4_ | 5600 (5 A g^−1^) | 98.7 | [23] |
| Zn//Co[Co_1_/4Fe_3_/4(CN)_6_]-I_2_ with PAM | 2000 (4 A g^−1^) | 100 | [24] |
| Zn//ACF-I_2_ with ZnSO_4_ | 3000 (2 C) | 97 | [25] |
| Zn//MX-AB@I with ZnSO_4_ and Li_2_SO_4_ | 500 (1 A g^−1^) | 91.1 | [26] |
| Zn//CuPc@rGO-ZnI_2_ with ZnSO_4_ | 1000 (3 A g^−1^) | 88.8 | [27] |
| Zn//LA133@I_2_ with ZnSO_4_ and Li_2_SO_4_ | 2700 (2 A g^−1^) | 80.9 | [28] |
| Zn//I_2_@MBene-Br with Zn(OTf)_2_ and LiTFSI | 3000 (1 A g^−1^) | 61.7 | [29] |
| **Zn//I_2_ with PASHE** | **2600 (1 A g^−1^)**  **9000 (10 A g^−1^)** | **78.9**  **94.9** | **This Work** |

**References**

1. Z. Li, W. Cao, T. Hu, Y. Hu, R. Zhang et al., Deploying cationic cellulose nanofiber confinement to enable high iodine loadings towards high energy and high-temperature Zn-I_2_ battery. Angew. Chem. Int. Ed. **136**, e202317652 (2024). https://doi.org/10.1002/ange.202317652
2. K. Zheng, C. Zheng, L. Zhu, B. Yang, X. Jin et al., Machine learning enabled reusable adhesion, entangled network-based hydrogel for long-term, high-fidelity EEG recording and attention assessment. Nano-Micro Lett. **17**, 281 (2025). https://doi.org/10.1007/s40820-025-01780-7
3. J. Su, X. Yin, H. Zhao, H. Yang, D. Yang et al., Temperature-dependent nucleation and electrochemical performance of Zn metal anodes. Nano Lett. **22**, 1549−1556 (2022). https://doi.org/10.1021/acs.nanolett.1c04353
4. Z. Li, C. Zhang, Y. Wang, T. Kou, X. Fei et al., Elucidating the role of porous Zn anode in improving Zn nucleation and growth behavior for aqueous Zn-ion batteries. ACS Nano **19**, 38875−38888 (2025). https://doi.org/10.1021/acsnano.5c17741
5. M. Wu, X. Wang, Y. Guo, H. Li, F. Zhang et al., Bidirectional ion-electric field synergy via in situ grown BiOCl/Bi heterostructure enabling ultra-stable zinc anodes across wide temperatures. Sci. Bull. **70**, 3774−3781 (2025). <https://doi.org/10.1016/j.scib.2025.10.004>
6. J. Hong, B. Zhu, M. Song, X. Wang, B. Gao et al., Fast ion transport interphase constructed by hollow mesoporous Na_3_V_2_(PO_4_)_3_ for stable zinc anode. Adv. Funct. Mater. **35**, 2424731 (2025). https://doi.org/10.1002/adfm.202424731
7. V.P. Nguyen, M. Park, Y.-W. Byeon, S. Lim, K. Yim et al., Ultrathin yet effective: 90 nm ZnF_2_ layer for stabilizing zinc-metal anodes. ACS Energy Lett. **10**, 5503−5511 (2025). https://doi.org/10.1021/acsenergylett.5c02565
8. C. Qiu, H. Huang, X. Zhu, L. Xue, M. Ni et al., Suppressing side reactions in spinel ZnMn_2_O_4_ for high-performance aqueous zinc-ion batteries. Energy Storage Mater. **75**, 104014 (2025). https://doi.org/10.1016/j.ensm.2025.104014
9. W. Shen, D. Chen, B. Song, W. Chen, Y. Zhang, et al., Cell to cell: A biomimetic hydrated deep eutectic electrolyte for wide-temperature zinc-ion capacitors. Energy Storage Mater. **82**, 104663 (2025). https://doi.org/10.1016/j.ensm.2025.104663
10. N. Gao, M. Cui, K. Xi, T. Deng, D. Yin et al., Elimination of concentration polarization under ultra-high current density zinc deposition by nanofluid self-driven ion enrichment. Adv. Mater. **37**, 2419034 (2025). https://doi.org/10.1002/adma.202419034
11. G. Liu, Y. Tang, Y. Wei, H. Li, J. Yan et al., Hydrophobic ion barrier-enabled ultradurable Zn (002) plane orientation towards long-life anode-less Zn batteries. Angew. Chem. Int. Ed. **63**, e202407639 (2024). https://doi.org/10.1002/anie.202407639
12. S. Wang, Y. Ying, S. Chen, H. Wang, K.K.K. Cheung et al., Highly reversible zinc metal anode enabled by zinc fluoroborate salt-based hydrous organic electrolyte. Energy Storage Mater. **63**, 102971 (2023). https://doi.org/10.1016/j.ensm.2023.102971
13. M. Zhou, Z. Luo, J. Lu, T. Xu, X. Zhung et al., Plane protection enabling (002) oriented plating and stripping processes for aqueous Zn-ion batteries. Energy Environ. Mater. **8**, e70056 (2025). https://doi.org/10.1002/eem2.70056
14. X. Zhao, J. Fu, M. Chen, Y. Wang, C. Huang et al., A self-phase separated electrolyte toward durable and rollover-stable zinc metal batteries. J. Am. Chem. Soc. **147**, 2714−2725 (2025). https://doi.org/10.1021/jacs.4c15132
15. Z. Xing, P. Ye, X. Shi, L. Shan, S. Guo et al., Design of cryogenic electrolyte with organic-free solvation structure for wide-temperature zinc metal batteries. Angew. Chem. Int. Ed. e202516974 (2025). https://doi.org/10.1002/anie.202516974
16. M. Tang, Q. Liu, X. Zou, Z. Yu, K. Zhang et al., Engineering in situ heterometallic layer for robust Zn electrochemistry in extreme Zn(BF_4_)_2_ electrolyte environment. Energy Storage Mater. **74**, 103896 (2025). https://doi.org/10.1016/j.ensm.2024.103896
17. J. Song, Z. Ren, Z. Chen, S. Zhang, Z. Yu et al., Inorganic and organic hybrid additives synergistically drive dual interface regulation to achieve highly stable aqueous zinc-ion batteries. Energy Storage Mater. **81**, 104523 (2025). https://doi.org/10.1016/j.ensm.2025.104523
18. J. Xu, Y. Yang, Q. Dai, Z. Zheng, Y. Cao et al., Towards ultra-stable wide-temperature zinc-ion batteries by using ion-sieving organic framework membrane. Angew. Chem. Int. Ed. **64**, e202423118 (2025). https://doi.org/10.1002/anie.202423118
19. Y. Yang, Q. He, C. Hu, X. Xie, S. Liang et al., Electron-initiated self-growth in situ hydrogel electrolyte with gradient protection interface enables stable zinc metal batteries. ACS Nano **19**, 21717−21728 (2025). https://doi.org/10.1021/acsnano.5c04942
20. Y. Yang, S. Guo, Y. Pan, B. Lu, S. Liang et al., Dual mechanism of ion (de)intercalation and iodine redox towards advanced zinc batteries. Energy Environ. Sci. **16**, 2358−2367 (2023). https://doi.org/ 10.1039/d3ee00501a
21. H. Zhao, D. Yin, Y. Qin, X. Cui, J. Feng et al., Highly electrically conductive polyiodide ionic liquid cathode for high-capacity dual-plating zinc−iodine batteries. J. Am. Chem. Soc. **146**, 6744−6752 (2024). https://doi.org/10.1021/jacs.3c12695
22. L. Zhang, M. Zhang, H. Guo, Z. Tian, L. Ge et al., A universal polyiodide regulation using quaternization engineering toward high value-added and ultra-stable zinc-iodine batteries. Adv. Sci. **9**, 2105598 (2022). https://doi.org/10.1002/advs.202105598
23. W. Liu, P. Liu, Y. Lyu, J. Wen, R. Hao et al., Advanced Zn–I_2_ battery with excellent cycling stability and good rate performance by a multifunctional iodine host. ACS App. Mater. Interfaces **14**, 8955−8962 (2022). https://doi.org/10.1021/acsami.1c21026
24. L. Ma, Y. Ying, S. Chen, Z. Huang, X. Li et al., Electrocatalytic iodine reduction reaction enabled by aqueous zinc-iodine battery with improved power and energy densities. Angew. Chem. Int. Ed. **60**, 3791−3798 (2021). https://doi.org/10.1002/ange.202014447
25. H. Pan, B. Li, D. Mei, Z. Nie, Y. Shao et al., Controlling solid–liquid conversion reactions for a highly reversible aqueous zinc-iodine battery. ACS Energy Lett. **2**, 2674−2680 (2017). http://pubs.acs.org/journal/aelccp
26. D. Li, Y.-J. Zhu, L. Cheng, S. Xie, H.-P. Yu et al., A MXene modulator enabled high-loading iodine composite cathode for stable and high-energy-density Zn-I_2_ battery. Adv. Energy Mater. **15**, 2404426 (2024). https://doi.org/10.1002/aenm.202404426
27. J. Kang, C. Wang, Z. Liu, L. Wang, Y. Meng et al., Electron-outflowing heterostructure hosts for high-voltage aqueous zinc-iodine batteries. Energy Storage Mater. **68**, 103367 (2024). https://doi.org/10.1016/j.ensm.2024.103367
28. K. Wang, H. Li, Z. Xu, Y. Liu, M. Ge et al., An iodine-chemisorption binder for high-loading and shuttle-free Zn–iodine batteries Adv. Energy Mater. **14**, 2470073 (2024). https://doi.org/10.1002/aenm.202470073
29. Z. Zhang, Y. Li, F. Mo, J. Wang, W. Ling et al., MBene with redox-active terminal groups for an energy-dense cascade aqueous battery. Adv. Mater. **36**, 2311914 (2024). https://doi.org/10.1002/adma.202311914
